# An expedient, mild and aqueous method for Suzuki–Miyaura diversification of (hetero)aryl halides or (poly)chlorinated pharmaceuticals†‡

**DOI:** 10.1039/d1qo00919b

**Published:** 2021-08-19

**Authors:** Sunil V. Sharma, Cristina Pubill-Ulldemolins, Enrico Marelli, Rebecca J. M. Goss

**Affiliations:** School of Chemistry and BSRC, University of St Andrews St Andrews KY16 9ST UK rjmg@st-andrews.ac.uk svs4@st-andrews.ac.uk

## Abstract

The development of mild, aqueous conditions for the cross-coupling of highly functionalized (hetero)aryl chlorides or bromides is attractive, enabling their functionalization and diversification. Herein, we report a general method for Suzuki–Miyaura cross-coupling at 37 °C in aqueous media in the presence of air. We demonstrate application of this general methodology for derivatisation of (poly)chlorinated, medicinally active compounds and halogenated amino acids. The approach holds the potential to be a useful tool for late-stage functionalization or analogue generation.

## Abstract 2

Simple, aqueous and direct cross-coupling of diverse and complex (hetero)aromatic halides and active pharmaceutical agents.

## Graphical abstract

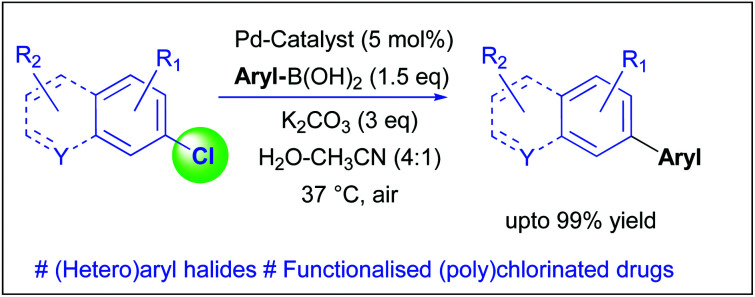

## Introduction

Palladium-catalysed cross-coupling reactions have become indispensable methodologies for the formation of C–C and carbon–heteroatom bonds under mild conditions with a wide range of organic halide and nucleophilic reagents. Palladium-catalysed cross-coupling reactions have largely utilised traditional organic solvents; the standard conditions for the Suzuki–Miyaura coupling typically require an organic, or mixed organic/aqueous base solvent system.^1,2^ The first example of a water-soluble palladium catalyst for cross-coupling reactions was reported by Casalnuovo.^3^ Significant effort has been devoted to the development of hydrophilic palladium/ligand complexes for aqueous-phase catalysis since this seminal work. In recent years, there has been increasing interest in the use of aqueous conditions in which hydrophobic catalysts are used with water as the reaction medium, as well as ligand-free catalysts based on palladium nanoparticles.^4–7^ The development of mild and functionally tolerant cross-coupling conditions for the modulation of challenging compounds is attractive, enabling the diversification of bioactive compounds, natural products and peptides.

Since developing the first Suzuki–Miyaura cross-coupling of unprotected chloro-/bromo-tryptophans,^8^ we reported the aqueous methodologies for Sonogashira,^9^ Heck,^10^ Buckwald-Hartwig amination^11^ and ketoarylation^12^ coupling chemistries. We also showed that Suzuki–Miyaura modification could be utilised to modulate the fluorescence properties of tryptophans,^8^ which was further exploited as an assay to detect the halogenases enzyme activity by Sewald *et al.*^13^ Subsequently we used the aqueous cross-coupling procedures, in a GenoChemetics approach, for natural product derivatization using chloropacidamycin, which was biosynthetically generated by installing a halogenase into the pacidamycins producing bacteria, *Streptomyces coeruleorubidis*.^14^ The O'Connor group further exemplified this approach in the synthetic modification of plant natural products using chlorinated-alkaloids.^15^ Applications of Suzuki–Miyaura cross-coupling procedures, focused on halotryptophans, could be translated to the modulation of peptides.^16,17^ We also reported the generation and modification of new to nature halogenated natural products in living cells and subsequently cross-couple the halo-metabolites in living bacterial cultures.^18^

Such advanced applications of cross-coupling methodologies require development of operationally simple, mild, aqueous reactions effective at physiological temperatures and in the presence of air. Within this context, we have successfully demonstrated that Pd/^*S*^SPhos catalyst is effective for the coupling of chloroindoles with *p*-toluene boronic acid in aqueous solvent at 37 °C.^18^ These results prompted us to extend this study and establish its application for a wide variety of (hetero)aromatic coupling partners and for derivatization of medicinally useful (poly)chlorinated pharmaceuticals.

## Results and discussion

**Scheme 1:**
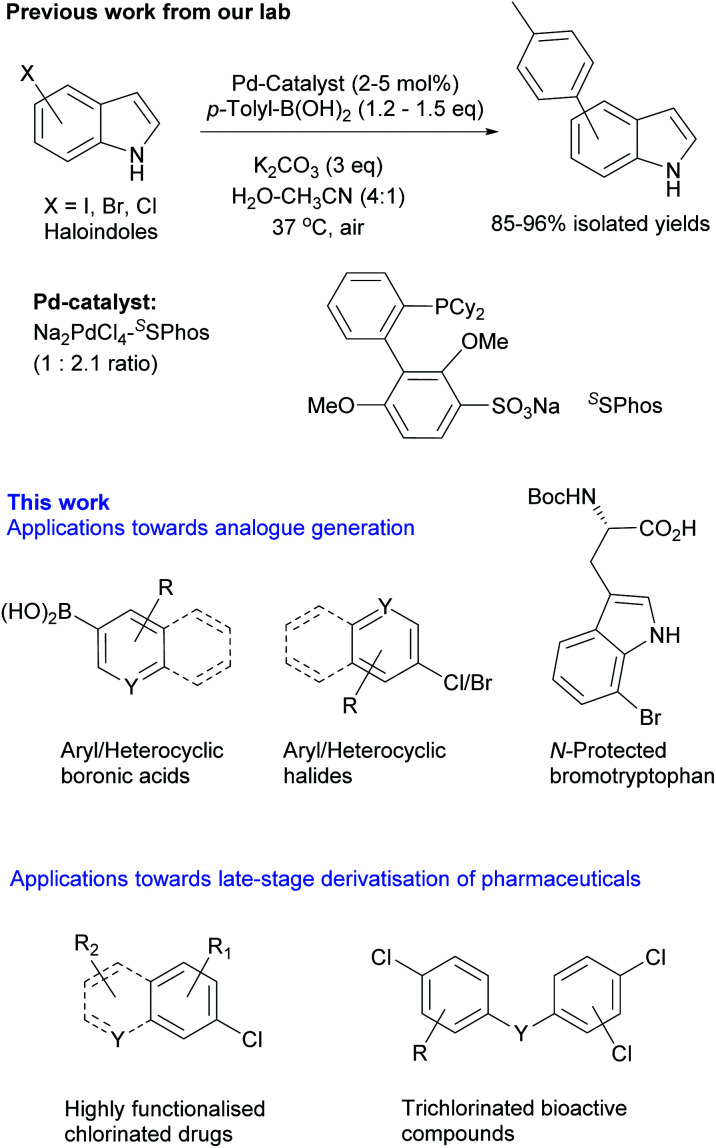
Aqueous, aerobic Suzuki–Miyaura cross-coupling methodology applied for derivatisation of various brominated/chlorinated (hetero)aryl compounds and chlorinated active pharmaceutical ingredients (APIs).

Initially, we focused on establishing water and air compatible reaction conditions for the cross-coupling of brominated and chlorinated indoles as model compounds. The indole heterocyclic moiety is a previleged motif, which is present in proteins in the form of the amino acid tryptophan, and in many bioactive compounds and natural products. There is some precedence for cross-coupling of haloindoles at mild temperatures, including the cross-coupling of 5-chloroindole at 40 °C in degassed THF.^19^ However, we aimed to establish a mild and general method for Suzuki–Miyaura cross-coupling under aqueous-aerobic conditions that is suitable for derivatization of unprotected heterocyclic and highly functionalized bioactive compounds. In this context, after systematically screening for the Pd-source/ligand/base/co-solvent, we identified that a combination of water-soluble palladium salt (Na_2_PdCl_4_) with readily available sulphonated phosphine ligand (^*S*^SPhos) and K_2_CO_3_ base was optimal for cross-coupling of haloindoles with *p*-tolyl-B(OH)_2_ in 4 : 1 water-acetonitrile mixture at 37 °C (Scheme 1 previous work^18^ and see Table S1, ESI‡ for details). The ligand was essential, no reaction was observed for 5-bromo/chloroindole using the Pd-salt without ^*S*^SPhos. This indicated a critical role of ^*S*^SPhos towards activation of Pd as well as subsequent oxidative addition under mild reaction conditions.^20^ Due to relatively poor aqueous solubility, (hetero)aryl chlorides formed a suspension in water-acetonitrile (4 : 1). Addition of Pd-^*S*^SPhos solution resulted in a cloudy non-homogenous mixture. Upon completion of the reaction, a black precipitate was always observed. When analysed, reactions in which precipitation did not occur were seen to have afforded no product. Based on these observations, we propose that the reaction is initiated by Pd-^*S*^SPhos akin to homogenous catalysis – however, during the course of reaction, we speculate formation of mixed Pd-species that play a role in catalysis.^21^ As our objective was catalytic utility, rather that determining the exact mechanism of the reactions, we further explored the synthetic utility of these mild conditions to diversify a wide range of scaffolds.

### Scope of (hetero)aryl boronic acids

Heterocyclic structures not only privileged compounds, but are often key fragments of many biologically active molecules. Many alkaloid natural products, such as reserpine (antihypertensive), serotonin (neurotransmitter), vinblastine (anticancer), contain the indole moiety.^22^

Several clinically useful marketed drugs containing indoles include indomethacin (anti-inflammatory), delavirdine (anti-HIV) and panobinostat (anticancer).^23^ To establish the general applicability of our cross-coupling conditions, we explored the reaction scope of a range of aryl boronic acids using 5-bromoindole as the halogenated heterocyclic substrate.

**Scheme 2:**
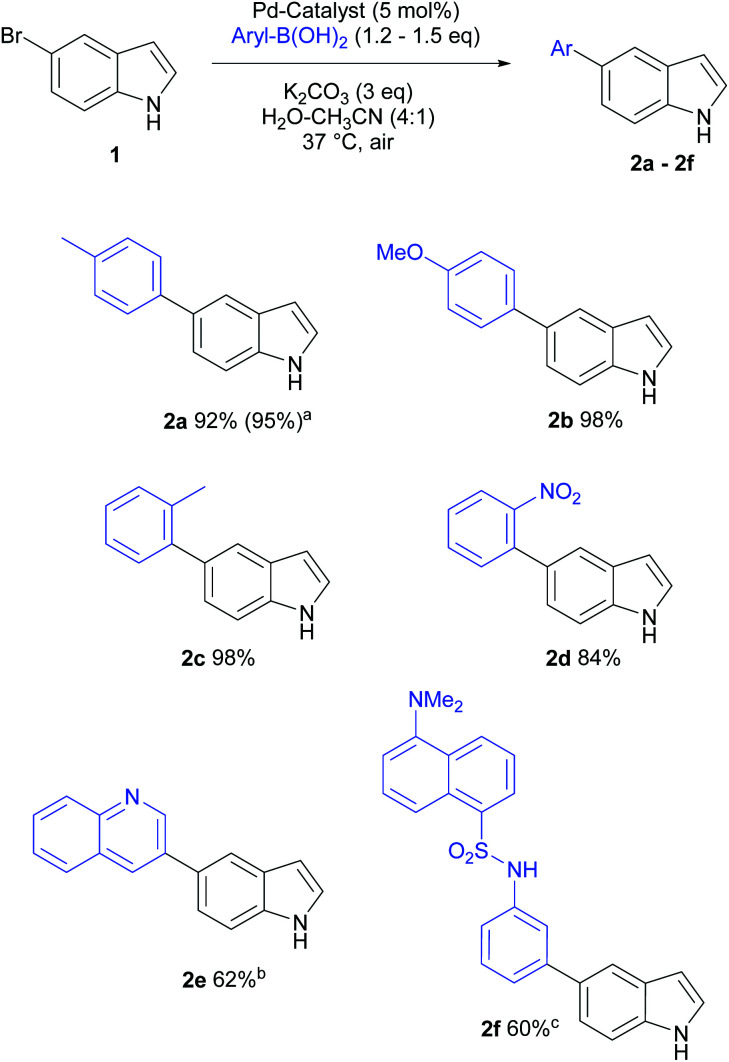
Scope of aryl boronic acids in Suzuki–Miyaura cross-coupling with 5-bromoindole. Reaction conditions: Haloindole (0.1 mmol), appropriate ArB(OH)_2_ (0.12–0.15 mmol), K_2_CO_3_ (0.3 mmol), Pd/^*S*^Sphos (5 mol%) in water–acetonitrile (4 : 1, 1 mL), 37 °C, 18 h. Isolated yields are reported after flash chromatography. ^*a*^Yield from 2 mmol scale reaction; ^*b*^two chromatographic purification steps required to isolate pure product; ^*c*^ reaction was carried out for 36 h.

Unprotected haloindoles are often challenging substrates for Pd-catalysed cross-coupling reactions due to free N–H groups, while suffering from a drawback of lower isolated yields due to potential side reactions (intermolecular coupling between NH and C–X) producing oligomers.^11,12^ It is noteworthy that this problem was not observed using our mild, aqueous Suzuki–Miyaura protocol in this study. In most cases, full conversions were observed, and lower isolated yield for **2e** and **2f** can be accounted to loss of material during chromatographic purifications. A variety of aryl boronic acids were well tolerated, affording 60–98% isolated yields of the target cross-coupling products (Scheme 2).

The reactions with electron-rich (*p*-methoxyphenyl, *p*-tolyl) as well as sterically encumbered (*o*-tolyl) boronic acids proceeded well (>92% isolated yields, **2a–2c**). The reaction with electron-deficient and generally less favoured *o*-nitrophenylboronic acid worked well (84% yield, **2d**). Other challenging coupling partner, quinoline-3-boronic acid, afforded the cross-coupling product **2e** in 62% yield. This low recovery of desired product was attributed to relatively poor solubility of heterocyclic boronic acid in mild, aqueous system as well as the need to repeat chromatographic purification steps. On the other hand, the reaction using fluorescent coupling partner, dansyl-3-aminophenylboronic acid (dansyl: [3-[5-(dimethylamino)naphthalen-1-yl]]), was found to be sluggish and 85% NMR yield was observed for **2f** after 36 h at 37 °C, whereas the isolated yield was moderate (60%). This method was generally applicable for cross-coupling of unprotected indolic halides with various aryl boronic acids and the fluorescence tagging of aryl halides under very mild conditions.

### Substrate scope: coupling of various (hetero)aromatic aryl halides

**Scheme 3:**
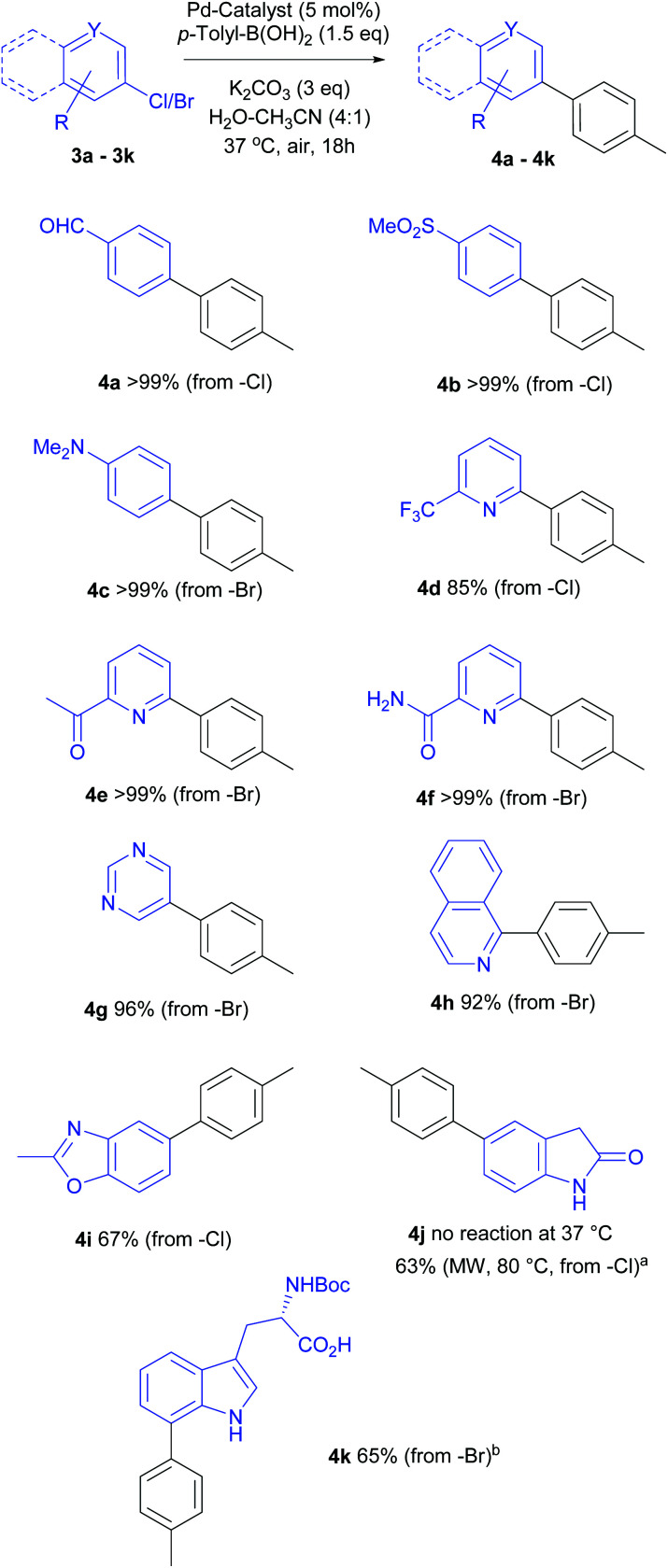
Scope of aryl halides in Suzuki–Miyaura cross-coupling with *p*-tolyl boronic acid. Reaction conditions: Aryl halide (0.1 mmol), *p*-tolyl-B(OH)_2_ (0.15 mmol), K_2_CO_3_ (0.3 mmol), Pd/^*S*^Sphos (5 mol%) in water-acetonitrile (4 : 1, 1 mL), 37 °C, 18 h. Isolated yields are reported after flash chromatography. ^*a*^No conversion was observed at 37 °C. Reported yield for the reaction conducted for 1 h at 80 °C using microwave heating; ^*b*^ Reaction was conducted at 37 °C for 24 h using *p*-tolyl-B(OH)_2_ (3 equiv.) and K_2_CO_3_ (6 equiv.).

Next, we turned our attention to the scope of aryl halides as coupling partners. As depicted in Scheme 3, a range of aryl, heteroaryl bromides or chlorides were successfully coupled with *p*-tolyl boronic acid to deliver the corresponding biaryl compounds. Most reactions gave full conversion after overnight stirring at 37 °C. In general, 1.5 equiv. (not optimized for each substrate) *p*-tolyl-B(OH)_2_ sufficed to achieve full conversion.

The mild, aqueous catalytic conditions were tolerant to a variety of functional groups, heterocycles and N-protected tryptophan substrates. Activated aryl chloride possessing electron-withdrawing groups (*p*-formyl or methylsulfoxide, **4a**, **4b**) as well as deactivated aryl bromide (with electron-donating *p*-dimethylamino group, **4c**) worked well and the corresponding products were isolated in quantitative yields.

Pyridine is a common heterocyclic motif found in pharmaceutically active compounds and natural products, hence, mild synthetic methods for pyridine and heterocyclic derivatives are desirable.^24^ Our reaction protocol translated well to the cross-coupling of various chloro-/bromo-pyridines elaborated with acetyl or carboxamido substituents, providing near quantitative yields of the target products (**4e**, **4f**); whilst the cross-coupling of trifluoromethyl substituted pyridyl-chloride resulted in an excellent yield of 85% (**4d**).

Consistent with the above results, 1-bromoisoquinoline and 5-bromopyrimidine afforded the coupling products **4h** (92%) and **4g** (96%) respectively. Disappointingly, under these mild reaction conditions, no coupling was observed for the very electron rich 5-chloro-2-oxyindole (**4j**) at 37 °C even after prolonged reaction times. However, a satisfying 63% yield of 5-(*p*-tolyl)-2-oxyindole was obtained after microwave heating for 1 h at 80 °C.

A literature search (Scifinder) did not reveal any published report of the Suzuki–Miyaura coupling of free 5-chloro-2-oxyindole, and the oxidative addition is likely to be particularly demanding, or unattainable at 37 °C for such a substrate. On the other hand, good conversion was observed for the coupling of 6-chloro-2-methylbenzoxazole, albeit, relatively lower yield (**4i**, 67%) was isolated. Considering the electron rich nature of benzoxazolyl chloride, and mild reaction conditions at 37 °C, this result is very encouraging and demonstrates a wide applicability of our protocol.

The Pd-mediated coupling of unprotected halotryptophans is challenging due to solubility issues, as well as the chelation of the catalyst.^18^ Our attempts to cross-couple the fully unprotected 5- or 7-bromotryptophan, using this general protocol, were unsuccessful. To achieve the desired reaction for this challenging substrate, we employed N-protected bromotryptophan. Indeed, repeating the reaction with *N*-boc-7-bromotrytpophan, a full conversion was observed within 24 h using 3 equiv. *p*-Tol-B(OH)_2_ and the product **4k** was isolated in a respectable 65% yield. This lower isolated yield could be due to purification challenges. All these examples indicate the versatile applications of this mild cross-coupling protocol.

### Direct cross-coupling of chloroarenes in pharmaceuticals and bioactive (poly)chlorides

To determine the applicability of this catalytic system on increasingly challenging substrates, an explorative study on more elaborate substrates was undertaken. Many over-the-counter drugs (OTC drugs) possess aryl chloride or heteroaryl chloride motif. We applied our mild cross-coupling protocol for pharmaceutically relevant chlorinated compounds and drugs. Using our optimised conditions, several OTC drugs were successfully converted to corresponding cross-coupling products in high to excellent yields.

**Scheme 4:**
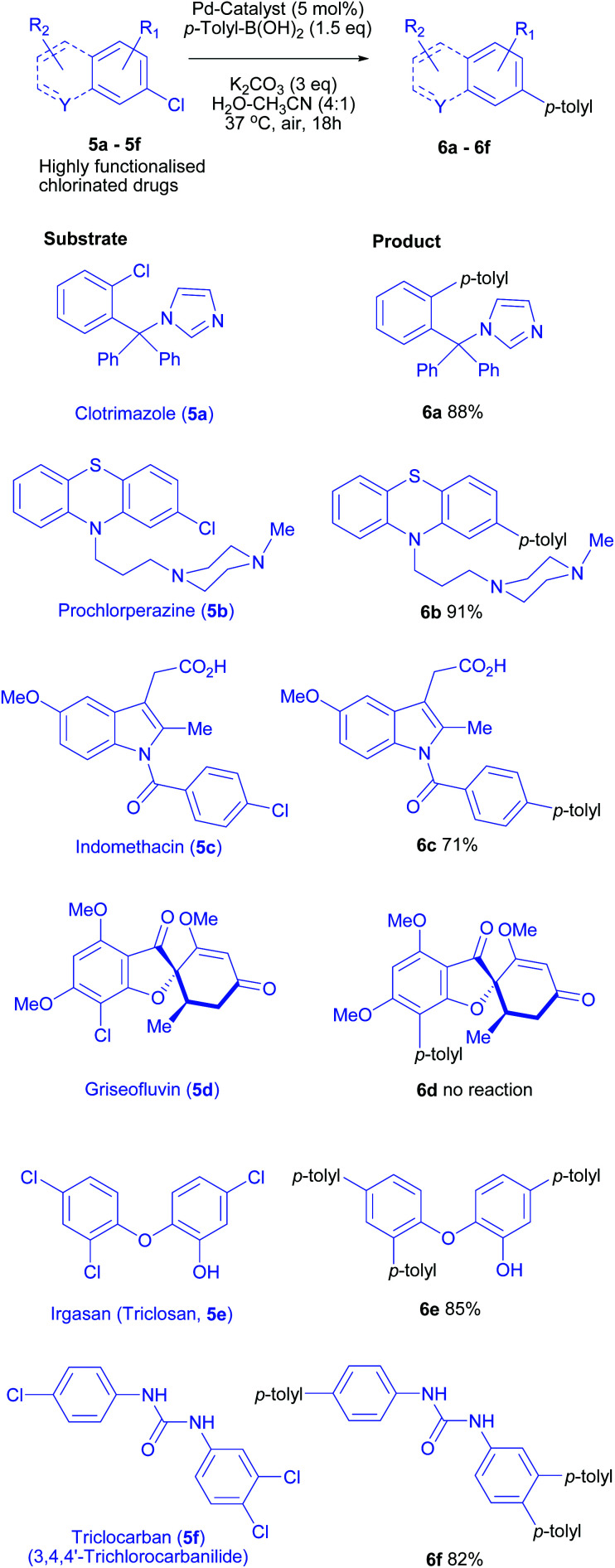
Suzuki–Miyaura cross-coupling of (poly)chlorinated over-the-counter drugs and medicinal agents. Reaction conditions: Aryl chloride (0.1 mmol), *p*-tolyl-B(OH)_2_ (0.15 mmol/Cl), K_2_CO_3_ (0.3–0.6 mmol), Pd/^*S*^Sphos (5 mol%) in water-acetonitrile (4 : 1, 1 mL), 37 °C, 18 h (36 h for trichloro substrates). Isolated yields are reported after flash chromatography. No conversion was observed for **5d** at 37 °C even after prolonged time.

The structural diversity and functional group complexity of all the chlorinated aromatic substrates screened is evident from Scheme 4.

Clotrimazole (**5a**, an azole antifungal possessing benzylic imidazole ring), prochlorperazine (**5b**, dopamine antagonist containing chlorophenothiazine heterocycle with a side chain piperidine) and indomethacin (**5c**, non-steroidal anti-inflammatory drug, an indole analogue with a free carboxylic acid) were successfully functionalised and high isolated yields (88, 91 and 71% respectively, **6a–6c**, Scheme 4) were obtained under mild conditions. The methodology was further challenged by testing against irgasan (**5e**, antimicrobial), an electron-rich trichlorinated phenolic ether. Gratifyingly, irgasan successfully underwent coupling at each of chlorides (85% isolated yield) using 4.5 equiv. *p*-Tol-B(OH)_2_. However, purification of these complex compounds was challenging due to co-elution/complexation of homocoupled by-product (*p*,*p*′-bitoluene), presumably due to pi-pi stacking with polyarylated products.

For the challenging chloroarylurea substrate **5f**, very few Pd-catalysed Suzuki–Miyaura cross-couplings are known. These protocols required either high temperature in organic solvents (90–150 °C), or elaborate degassing and tedious reaction set-up or use bespoke (non-commercial) catalysts.^25,26^

Using the reaction conditions developed in this study, a complete conversion was obtained for triclocarban (**5f**, antimicrobial, an aryl trichloride possessing a urea motif) and the tris-coupling product **6f** was isolated in 82% yield, proving the utility of this mild protocol for complex transformations. Unfortunately, sterically demanding and highly electron-rich aryl chloride (griseofulvin) failed to afford any cross-coupling product. Only unreacted starting material was recovered indicating lack of oxidative addition under the mild reaction conditions. Only 3 examples of Suzuki–Miyaura elaboration of griseofulvin are reported under forcing conditions at temperatures >100 °C.^27–29^ Regardless, above results highlight the strength of our mild cross-coupling methodology towards the potential applications for functionalisation of medicinal compounds.

## Conclusions

In conclusion, we report a straightforward and mild protocol for the Suzuki–Miyaura cross-coupling of various (hetero)aryl chlorides/bromides, including N-protected halotryptophan with a variety of aryl boronic acids. The cross-couplings were achieved in aqueous conditions, in the presence of air, at 37 °C. Importantly, these conditions could also be applied successfully to the modification of highly functionalized (poly)chlorinated pharmaceuticals, potentially opening ways towards the direct cross-coupling of halogenated molecules, using commercially available catalyst, under exceptionally mild conditions.

## Conflicts of interest

There are no conflicts to declare.

## Supplementary Material

QO-008-D1QO00919B-s001
