# Supplementary material for: An expedient, mild and aqueous method for Suzuki–Miyaura diversification of (hetero)aryl halides or (poly)chlorinated pharmaceuticals
Source: Org Chem Front. 2021 Aug 19;8(20):5722–7. doi: 10.1039/d1qo00919b (PMC8506956; doi:10.1039/d1qo00919b)
Supplement: QO-008-D1QO00919B-s001 [file QO-008-D1QO00919B-s001.pdf]

Electronic Supplementary Material (ESI) for Organic Chemistry Frontiers.
This journal is © the Partner Organisations 2021

Electronic Supplementary Information

An expedient, mild and aqueous method for Suzuki-Miyaura diversification of (hetero)aryl halides or (poly)chlorinated pharmaceuticals†

Sunil V. Sharma, Cristina Pubill-Ulldemolins, Enrico Marelli and Rebecca J. M. Goss

Contents:

	Page
General experimental	2
Table S1: Initial screening of Pd-catalysts and bases for Suzuki-Miyaura cross-coupling for various 5-haloindoles with *p*-Tol-B(OH)$_2$	3
General procedure for Suzuki-Miyaura cross-coupling reaction	3
Characterisation data of purified products	3-8
References	8
NMR spectra	9-31

General Experimental

All reagents were purchased from commercial suppliers and were used without further purification unless otherwise stated. Proton NMR (^{1}H), carbon NMR (^{13}C) and fluorine NMR (^{19}F) were recorded on either a Bruker Ascend 500 (500 MHz), Bruker 500 UltraShield (500 MHz), Bruker 400 UltraShield (400 MHz) or a Bruker UltraShield (300 MHz) spectrometer. The NMR experiments were carried out in deuterochloroform ($CDCl_3$) or deuterated methanol (d_4-MeOH). The chemical shifts (δ) are quoted in parts per million (ppm). Using a DEPT-Q sequence, the ^{13}C NMR spectra are depicted to indicate CH_3/ CH and CH_2 / $C_{quaternary}$ in opposite phase. Multiplicities are abbreviated as s, singlet; d, doublet; t, triplet; q, quartet; m, multiplet; b, broad for the ^{1}H NMR spectra. Coupling constants are reported in Hertz (Hz).

Column chromatography was performed using Davisil silica gel LC60A (40-63 micron). Flash column chromatography was performed on a Biotage Isolear-4 using snap-silica cartridges. Thin layer chromatography (TLC) was performed using aluminium sheets of silica gel 60 F254 and was visualised under a Mineralight model UVGL-58 lamp (254 nm). The plates were developed with basic potassium permanganate solutions.

High- and low-resolution mass spectra were recorded at the University of St Andrews on a Waters Micromass LCT mass spectrometer coupled to a Waters 2975 HPLC system; or on an Orbitrap Velos pro or at the EPSRC National Mass Spectrometry Service, Swansea. Microwave reactions were conducted in sealed vials using a Biotage Initiator+ microwave reactor.

7-Bromotryptophan and *N*-Boc-7-bromotryptophan were prepared as described previously.[1] Optimisation of these reaction conditions were detailed in our earlier publication.[2] Along with water, to improve the solubility of substrates, acetonitrile was found beneficial organic co-solvent (4:1 water-acetonitrile ratio). A series of readily available sulphonated water soluble phosphine ligand (including TPPTS, ^{S}SPhos, ^{S}XPhos) in combination with Pd-salts such as Na_2PdCl_4, $Pd(OAc)_2$ and $PdCl_2$ were screened. In addition to these systems, we also evaluated pre-catalysts based on bidentate ligands such as [$PdCl_2$(dppf)], [$PdCl_2$(dtbpf)], [$PdCl_2$(Xantphos)] and NHC-Pd complexes. Mild bases (K_2CO_3, Na_2CO_3, K_3PO_4, Cs_2CO_3) were also screened. In this preliminary screen, for the cross-coupling 5-iodo- or 5-bromoindole with *p*-tolyl boronic acid (*p*-Tol-B$(OH)_2$); the use of the water-soluble Na_2PdCl_4 catalyst in combination with ^{S}SPhos ligand, and K_2CO_3 as base were found to be the most suitable. These optimised aqueous conditions were applied to diversify a range of halogenated aromatic compounds and aryl boronic acids in this study.

Table S1. Initial screening of Pd-catalyst and bases for Suzuki-Miyaura cross-coupling for various 5-haloindoles with *p*-Tol-B(OH)$_2$

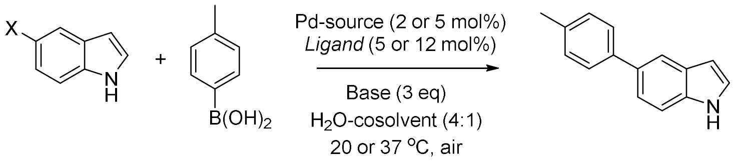

	X=	Pd-Ligand (1:2.5 ratio)	Base	Solvent	Time (Temp.)	Conversion (%)[a]
**Catalyst screen**						
	Br	Na_2PdCl_4-^{S}SPhos (5 mol%)	K_2CO_3	Water- CH_3CN (4:1)	18 h (37 °C)	99
	Br	Na_2PdCl_4-^{S}XPhos (5 mol%)	K_2CO_3	Water- CH_3CN (4:1)	18 h (37 °C)	20
	Br	Na_2PdCl_4-TPPTS (5 mol%)	K_2CO_3	Water- CH_3CN (4:1)	18 h (37 °C)	48
	Br	(dtbpf)$PdCl_2$ (5 mol%)	K_2CO_3	Water- CH_3CN (4:1)	18 h (37 °C)	65
	Br	Xantphos.$PdCl_2$ (5mol%)	K_2CO_3	Water- CH_3CN (4:1)	18 h (37 °C)	25
**Base screen**						
	I	Na_2PdCl_4-^{S}SPhos (2 mol%)	K_2CO_3	Water- CH_3CN (4:1)	8 h (37 °C)	98
	I	Na_2PdCl_4-^{S}SPhos (2 mol%)	Na_2CO_3	Water- CH_3CN (4:1)	8 h (37 °C)	58
	I	Na_2PdCl_4-^{S}SPhos (2 mol%)	Cs_2CO_3	Water- CH_3CN (4:1)	8 h (37 °C)	70
	I	Na_2PdCl_4-^{S}SPhos (2 mol%)	Na_3PO_4	Water- CH_3CN (4:1)	8 h (37 °C)	85

Conditions: A mixture of 5-haloindole (0.1 mmol), Pd-catalyst (2 or 5 mol%), ligand (5 or 12 mol%), *p*-Tol-B(OH)$_2$ (0.15 mM) and appropriate base (0.3 mmol) in corresponding solvent (2 ml) was stirred at specified temperature. [a]Conversion was determined by ^{1}H NMR of the crude reaction.

General Procedure for mild, aqueous Suzuki-Miyaura cross-coupling

In a screw cap glass vial, appropriate aryl halide (0.1 mmol), arylboronic acid (0.15 mmol), potassium carbonate (42 mg, 0.3 mmol) were suspended in water-CH_3CN mixture (4:1, 1.8 mL). A solution of Na_2PdCl_4 (5 mol%, 1.4 mg) and ^{S}SPhos (12 mol%, 6 mg) in water (0.2 mL) was added. The vial was closed and stirred at 37 °C until complete consumption of aryl halide was observed by TLC. The reaction was diluted with brine (2 mL) and extracted with ethyl acetate (3 × 3-4 mL). Combined organic extract was washed with brine, dried ($MgSO_4$), filtered and solvent removed under reduced pressure. The desired product was isolated by flash chromatography using dichloromethane or ethyl acetate in hexanes (5-100% gradient).

Characterisation data of purified products

2a: 5-(*p*-Tolyl)-1H-indole

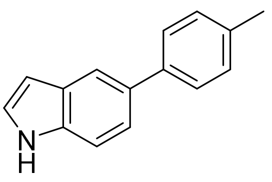

The general procedure afforded 19 mg (92% from 5-bromoindole) of the desired product as a white solid. **^{1}H NMR** (300 MHz, $CDCl_3$) : δ 8.12 (bs, 1H), 7.95 – 7.88 (bs, 1H), 7.63 (d, *J* = 8.1 Hz, 2H), 7.53 – 7.42 (m, 2H), 7.32 (d, *J* = 8.1 Hz, 2H), 7.27 – 7.22 (m, 1H), 6.70 – 6.62 (m, 1H), 2.47 (s, 3H) ppm; **^{13}C NMR** (75 MHz, $CDCl_3$) : δ 139.78, 136.07, 135.26, 133.46, 129.50, 128.47, 127.35, 124.91, 121.93, 119.09, 111.30, 103.05, 21.19 ppm; **MS (ESI)** *m/z* 208.09 [M+H]$^+$; **HRMS (FTMS +p ESI)** *m/z* $C_{15}H_{14}N$

$[M+H]^+$ calculated 208.1121, found 208.1120. NMR data was in agreement with literature reported data.[3]

2b: 5-(*p*-Methoxyphenyl)-1H-indole.

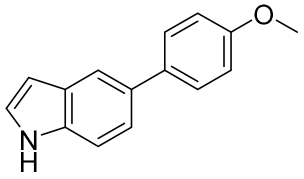

The general procedure afforded 22 mg (98%) of the desired product as a white waxy solid. **^{1}H NMR** (400 MHz, $CDCl_3$): δ 8.19 (bs, 1H), 7.89 – 7.84 (m, 1H), 7.66 – 7.59 (m, 2H), 7.50 – 7.42 (m, 2H), 7.27 (dd, *J* = 3.1, 2.5 Hz, 1H), 7.04 (d, *J* = 8.8 Hz, 2H), 6.68 – 6.61 (m, 1H), 3.91 (s, 3H) ppm; **^{13}C NMR** (100 MHz, $CDCl_3$) : δ 158.6, 135.3, 135.1, 133.2, 128.5, 128.5, 124.9, 121.8, 118.9, 114.3, 111.4, 103.0, 55.5 ppm; **MS (ESI)** *m/z* 224.09 $[M+H]^+$; **HRMS (FTMS +p ESI)** *m/z* $C_{15}H_{14}NO$ $[M+H]^+$ calculated 224.1070, found 224.1069. NMR data was in agreement with literature reported data.[3]

2c: 5-(*o*-Tolyl)-1H-indole.

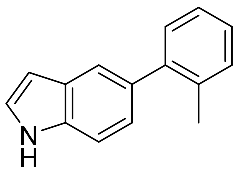

The general procedure afforded 20 mg (98%) of the desired product as an off-white solid. **^{1}H NMR** (400 MHz, $CDCl_3$) : δ 8.22 (bs, 1H), 7.66 – 7.60 (m, 1H), 7.47 (dt, *J* = 8.4, 0.8 Hz, 1H), 7.40 – 7.25 (m, 5H), 7.22 (dd, *J* = 8.3, 1.6 Hz, 1H), 6.63 (ddd, *J* = 2.1, 1.1, 0.9 Hz, 1H), 2.36 (s, 3H) ppm; **^{13}C NMR** (100 MHz, $CDCl_3$) : δ 143.2, 135.9, 134.9, 133.9, 130.5, 130.3, 127.9, 126.8, 125.7, 124.7, 123.9, 121.2, 110.6, 102.9, 20.8 ppm; **MS (ESI)** *m/z* 208.08 $[M+H]^+$; **HRMS (FTMS +p ESI)** *m/z* $C_{15}H_{14}N$ $[M+H]^+$ calculated 208.1121, found 208.1122. NMR data was in agreement with literature reported data.[3]

2d: 5-(*o*-Nitrophenyl)-1H-indole.

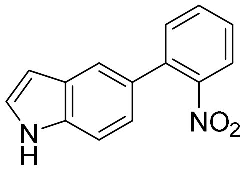

The general procedure afforded 20 mg (84%) of the desired product as a yellow solid. **^{1}H NMR** (400 MHz, $CDCl_3$) : δ 8.30 (s, 1H), 7.85 (dd, *J* = 8.1, 1.1 Hz, 1H), 7.69 – 7.65 (m, 1H), 7.63 (dd, *J* = 7.5, 1.2 Hz, 1H), 7.57 (dd, *J* = 7.7, 1.4 Hz, 1H), 7.53 – 7.39 (m, 2H), 7.33 – 7.25 (m, 1H), 7.17 (dd, *J* = 8.4, 1.7 Hz, 1H), 6.62 (ddd, *J* = 3.0, 2.0, 0.8 Hz, 1H) ppm; **^{13}C NMR** (100 MHz, $CDCl_3$) : δ 149.9, 137.5, 135.6, 132.6, 132.1, 129.0, 128.3, 127.5, 125.3, 123.9, 122.1, 120.3, 111.5, 103.2 ppm; **MS (ESI)** *m/z* 239.09 $[M+H]^+$, 261.09 $[M+Na]^+$; **HRMS (FTMS +p ESI)** *m/z* $C_{14}H_{11}N_2O_2$ $[M+H]^+$ calculated 239.0815, found 239.0814. NMR data was in agreement with literature reported data.[4]

2e: 5-(Quinolin-3-yl)-1H-indole.

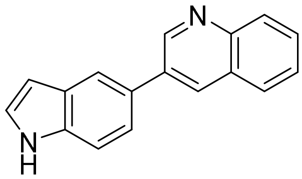

The general procedure afforded 16 mg (62%) of the desired product as a pale yellow oil. **^{1}H NMR** (400 MHz, $CDCl_3$) : δ 9.28 (d, *J* = 2.3 Hz, 1H), 8.36 (bs, 1H), 8.35 (d, *J* = 2.1 Hz, 1H), 8.15 (d, *J* = 8.5 Hz, 1H), 8.04 – 7.96 (m, 1H), 7.89 (dd, *J* = 8.1, 1.3 Hz, 1H), 7.71 (ddd, *J* = 8.4, 6.9, 1.5 Hz, 1H), 7.61 – 7.54 (m, 3H), 7.31 (dd, *J* = 3.1, 2.5 Hz, 1H), 6.68 (dd, *J* = 3.8, 1.4 Hz, 1H) ppm; **^{13}C NMR** (75 MHz, $CDCl_3$) : δ 150.7, 146.9, 135.8, 135.3, 133.1, 129.9, 129.2, 129.0, 128.8, 128.4, 128.03, 126.9, 125.5, 121.9, 119.9, 111.9, 103.3 ppm; **MS (ESI)** *m/z* 245.17 $[M+H]^+$; **HRMS (FTMS +p ESI)** *m/z* $C_{17}H_{13}N_2$ $[M+H]^+$ calculated 245.1073, found 245.1064.

2f: 5-[3-(5-Dimethylaminonaphthalene-1-sulfonylamino)]-1H-indole.

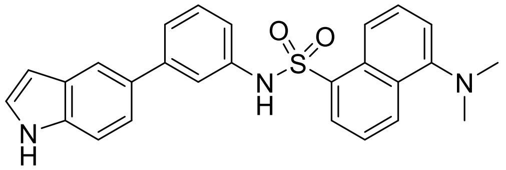

The general procedure afforded 26 mg (60%) of the desired product as a yellow solid. **^{1}H NMR** (400 MHz, $CDCl_3$) : δ 8.50 (dt, *J* = 8.5, 1.0 Hz, 1H), 8.39 (d, *J* = 8.7 Hz, 1H), 8.23 (dd, *J* = 7.4, 1.3 Hz, 1H), 7.63 – 7.54 (m, 2H), 7.44 (dd, *J* = 8.5, 7.4 Hz, 1H), 7.36 (dt, *J* = 8.4, 0.7 Hz, 1H), 7.30 (ddd, *J* = 7.8, 1.7, 1.1 Hz, 1H), 7.25 – 7.21 (m, 1H), 7.20 – 7.14 (m, 3H), 7.12 (t, *J* = 1.9 Hz, 1H), 6.90 (ddd, *J* = 8.0, 2.2, 1.0 Hz, 1H), 6.88 (s, 1H), 6.56 (ddd, *J* = 3.0, 2.0, 0.9 Hz, 1H), 2.86 (s, 6H) ppm; **^{13}C NMR** (75 MHz, $CDCl_3$) : δ 152.2, 143.7, 136.8, 135.5, 134.2, 132.3, 130.9, 130.6, 129.9, 129.8, 129.4, 128.8, 128.4, 125.1, 124.4, 123.3, 121.7, 120.7, 119.6, 119.3, 118.6, 115.4, 111.4, 103.0, 45.5 ppm; **MS (ESI)** *m/z* 442.17 $[M+H]^+$; **HRMS (FTMS +p ESI)** *m/z* $C_{26}H_{24}N_3O_2S$ $[M+H]^+$ calculated 442.1584, found 442.1570.

4a: 4'-Methyl-[1,1'-biphenyl]-4-carbaldehyde.

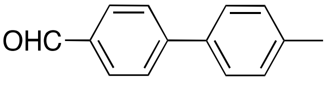

The general procedure afforded 19.6 mg (quantitative yield) of the desired product as a white solid. **^{1}H NMR** (400 MHz, $CDCl_3$) : δ 10.05 (s, 1H), 7.94 (d, *J*

= 8.3 Hz, 2H), 7.74 (d, J=8.3 Hz, 2H), 7.55 (d, *J* = 8.0, 2H), 7.29 (d, *J* = 8.0, 2H), 2.42 (s, 3H) ppm; **^{13}C NMR** (100 MHz, $CDCl_3$) : δ 191.9, 147.1, 138.5, 136.8, 134.9, 130.3, 129.7, 127.4, 127.2, 21.2 ppm; **HRMS (FTMS +p ESI)** *m/z* $C_{14}H_{13}O$ $[M+H]^+$ calculated 197.0961, found 197.0964. NMR data was in agreement with literature reported data. NMR data was in agreement with literature reported data.[5]

4b: 4-Methyl-4'-(methylsulfonyl)-1,1'-biphenyl.

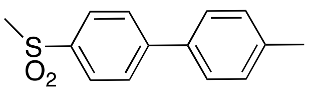

The general procedure afforded 24.6 mg (quantitative yield) of the desired product as a white solid. **^{1}H NMR** (400 MHz, $CDCl_3$) : δ 7.99 (dt, *J* = 8.8, 2 Hz, 2H), 7.76 (dt, *J* = 8.8, 2.0 Hz, 2H), 7.52 (dt, *J* = 8.4, 1.8 Hz, 2H), 7.30 (d, *J* = 8.0 Hz, 2H), 3.09 (s, 3H) 2.42 (s, 3H) ppm; **^{13}C NMR** (100 MHz, $CDCl_3$) : δ 146.6, 138.7, 138.7, 136.2, 129.8, 127.9, 127.7, 127.2, 44.6, 21.2 ppm; **HRMS (FTMS +p ESI)** *m/z* $C_{11}H_{11}N_2$ $[M+H]^+$ calculated 171.0917, found 171.0914. NMR data was in agreement with literature reported data.[6]

4c: *N*,*N*,4'-Trimethyl-[1,1'-biphenyl]-4-amine.

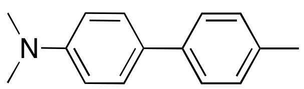

The general procedure afforded 21.1 mg (quantitative yield) of the desired product as a yellow solid. **^{1}H NMR** (400 MHz, $CDCl_3$) : δ 7.52-7.44 (m, 4H), 7.22 (d, *J* = 7.5 Hz, 2H), 6.85 (d, *J* = 8.2, 2H), 3.00 (s, 6H), 2.38 (s, 3H) ppm; **^{13}C NMR** (100 MHz, $CDCl_3$) : δ 146.6, 138.3, 135.7, 129.4, 127.6, 126.2, 113.2, 40.9, 21.0 ppm; **HRMS (FTMS +p ESI)** *m/z* $C_{15}H_{18}N$ $[M+H]^+$ calculated 212.1468, found 212.1434. NMR data was in agreement with literature reported data.[7]

4d: 2-(*p*-Tolyl)-6-(trifluoromethyl)pyridine.

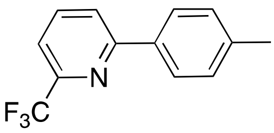

The general procedure afforded 20.2 mg (85% yield) of the desired product as a white solid. **^{1}H NMR** (400 MHz, $CDCl_3$) : δ 7.97 (d, J = 8.4 Hz, 2H), 7.90-7.87 (m, 2H), 7.60-7.55 (m, 1H), 7.30 (d, *J* = 8.0 Hz, 2H), 2.42 (s, 3H) ppm; **^{13}C NMR** (100 MHz, $CDCl_3$) : δ 157.8, 148.0 (q, *J* = 34 Hz), 139.9, 137.9, 135.0, 129.6, 126.9, 122.4, 121.8 (q, *J* = 273 Hz), 118.1 (q, *J* = 2.8 Hz), 21.3 ppm; **^{19}F NMR** (372 MHz, $CDCl_3$) : δ -68.1 ppm; **HRMS (FTMS +p ESI)** *m/z* $C_{13}H_{11}F_3N$ $[M+H]^+$ calculated 238.0838, found 238.0834. NMR data was in agreement with literature reported data.[8]

4e: 1-(6-(*p*-pyridin-2-yl)ethenone.

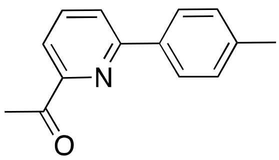

The general procedure afforded 21.1 mg (quantitative yield) of the desired product as a white solid. **^{1}H NMR** (400 MHz, $CDCl_3$) : δ 8.03 (dt, *J* = 8.0, 2.0 Hz, 2H), 7.97 (dd, *J* = 7.2, 1.6 Hz, 1H), 7.94-7.85 (m, 2H), 7.34 (d, *J* = 8.8 Hz, 2H), 2.85 (s, 3H) 2.46 (s, 3H) ppm; **^{13}C NMR** (125 MHz, $CDCl_3$) : δ 200.7, 156.4, 153.3, 139.5, 137.5, 135.6, 129.6, 126.7, 123.1, 119.4, 25.8, 21.3 ppm; **HRMS (FTMS +p ESI)** *m/z* $C_{14}H_{13}NO$ $[M+H]^+$ calculated 212.1070, found 212.1067. NMR data was in agreement with literature reported data.[9]

4f: 6-(*p*-Tolyl)picolinamide.

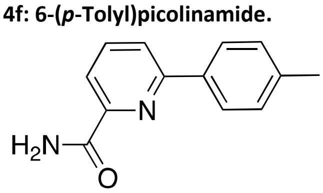

The general procedure afforded 21.2 mg (quantitative yield) of the desired product as a white solid. **^{1}H NMR** (400 MHz, $CDCl_3$) : δ 8.13 (dd, *J* = 7.2, 1.6 Hz, 2H), 8.04 (bs, 1H), 7.93-7.88 (m 3H), 7.85 (dd, *J* = 8.0, 1.2 Hz, 1H), 7.31 (d, *J* = 7.6 Hz, 2H), 6.03 (bs, 1H), 2.43 (s, 3H) ppm; **^{13}C NMR** (100 MHz, $CDCl_3$) : δ 167.0, 156.1, 149.1, 139.6, 138.0, 135.5, 129.5, 126.8, 122.9, 120.4, 21.3 ppm; **HRMS (FTMS +p ESI)** *m/z* $C_{13}H_{13}N_2O$ $[M+H]^+$ calculated 213.1022, found 213.1026.

4g: 5-(*p*-Tolyl)pyrimidine.

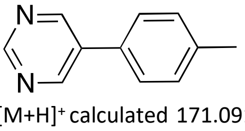

The general procedure afforded 16.2 mg (96% yield) of the desired product as an off-white solid. **^{1}H NMR** (400 MHz, $CDCl_3$) : δ 9.18 (s, 1H), 8.93 (s, 2H), 7.48 (d, *J* = 7.6, 2H), 7.32 (d, *J* = 7.6 Hz, 2H), 2.43 (s, 3H) ppm; **^{13}C NMR** (100 MHz, $CDCl_3$) : δ 157.2, 154.7, 139.1, 134.2, 131.3, 130.1, 126.8, 21.2 ppm; **HRMS (FTMS +p ESI)** *m/z* $C_{11}H_{11}N_2$ $[M+H]^+$ calculated 171.0917, found 171.0914. NMR data was in agreement with literature reported data. NMR data was in agreement with literature reported data.[10]

4h: 1-(*p*-Tolyl)isoquinoline.

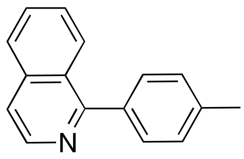

The general procedure afforded 20.2 mg (92% yield) of the desired product as a colorless oil. **^{1}H NMR** (500 MHz, $CDCl_3$) : δ 8.60 (d, *J* = 5.5 Hz, 1H), 8.14 (dd, *J* = 8.5, 1.0 Hz, 1H), 7.88 (d, *J* = 8.0 Hz, 1H), 7.88 (d, *J* = 8.0, 1H), 7.69 (ddd, *J* = 8.1, 6.9, 1.1 Hz, 1H), 7.64 (d, *J* = 6.0 Hz, 1H), 7.61 (d, *J* = 8.0 Hz, 2H), 7.54 (ddd, *J* = 8.2, 6.9, 1.1 Hz, 1H), 7.34 (d, *J* = 7.5 Hz, 2H) 7.35 (bs, 1H), 2.47 (s, 3H) ppm; **^{13}C NMR** (125 MHz, $CDCl_3$) : δ 160.8, 142.0, 136.8, 136.5, 129.9, 129.8, 129.0, 127.7, 127.1, 126.9, 126.7, 119.8, 21.4 ppm; **HRMS (FTMS +p ESI)** *m/z* $C_{16}H_{14}N$ $[M+H]^+$ calculated 220.1121, found 220.1117. NMR data was in agreement with literature reported data. NMR data was in agreement with literature reported data.[11]

4i: 2-Methyl-5-(*p*-tolyl)benzo[*d*]oxazole.

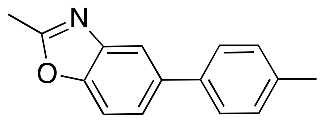

The general procedure afforded 14.9 mg (67% yield) of the desired product as a white solid. **^{1}H NMR** (400 MHz, $CDCl_3$) : δ 8.13 (dd, *J* = 7.2, 1.6 Hz, 2H), 8.04 (bs, 1H), 7.93-7.88 (m 3H), 7.85 (dd, *J* = 8.0, 1.2 Hz, 1H), 7.31 (d, *J* = 7.6 Hz, 2H), 6.03 (bs, 1H), 2.43 (s, 3H) ppm; **^{13}C NMR** (125 MHz, $CDCl_3$) : δ 163.8, 150.4, 142.1, 138.2, 137.9, 136.9, 129.5, 127.3, 123.9, 117.7, 110.1, 21.1, 14.6 ppm; **HRMS (FTMS +p ESI)** *m/z* $C_{15}H_{14}NO$ $[M+H]^+$ calculated 224.1070, found 224.1069.

4j: Synthesis of 5-(*p*-Tolyl)-2-oxindole using microwave heating.

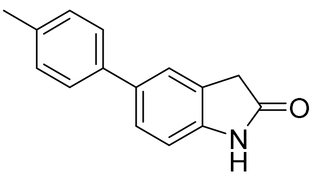

In a microwave vial, 5-chloro-2-oxindole (17 mg, 0.1 mmol), *p*-tolylboronic acid (20 mg, 0.15 mmol), potassium carbonate (42 mg, 0.3 mmol) were suspended in water-CH_3CN mixture (4:1, 1.5 mL). A solution of Na_2PdCl_4 (5 mol%, 1.4 mg) and ^{S}SPhos (12 mol%, 6 mg) in water (0.2 mL) was added. The vial was sealed with an aluminium crimp cap and heated at 80 °C for 1 h under microwave irradiation. After cooling, the reaction was diluted with water (2 mL) and extracted with ethyl acetate (3 × 3 mL). Combined organic extract was dried ($MgSO_4$), filtered and solvent removed under reduced pressure. Recrystallisation from MeOH afforded 14 mg (63% yield) of the desired product as a white solid. **^{1}H NMR (500 MHz, $CDCl_3$)** : δ 8.28 (s, 1H), 7.51 – 7.47 (m, 2H), 7.46 (d, *J* = 8.2 Hz, 2H), 7.26 (d, *J* = 7.9 Hz, 2H), 6.96 (d, *J* = 8.0 Hz, 1H), 3.63 (s, 2H), 2.42 (s, 3H) ppm; **^{13}C NMR (125 MHz, $CDCl_3$)** : δ 177.3, 141.4, 138.0, 136.8, 135.9, 129.6, 126.8, 126.7, 125.9, 123.5, 109.8, 36.3, 21.1 ppm; **MS (ESI)** *m/z* 224.09 $[M+H]^+$; **HRMS (FTMS +p ESI)** *m/z* $C_{15}H_{14}NO$ $[M+H]^+$ calculated 224.1070, found 224.1068.

4k: *N*-Boc-7-(*p*-tolyl)-*S*-tryptophan.

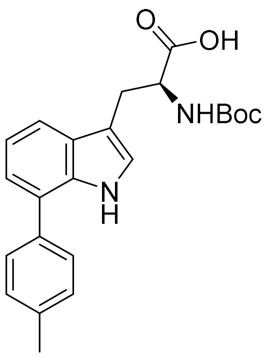

The general procedure afforded 12 mg (65% isolated yield) of the desired product as clear, colourless solid using *N*-Boc-7-bromo-*S*-tryptophan (18 mg, 0.047 mmol), *p*-tol-$B(OH)_2$ (20 mg, 3 equiv.) and K_2CO_3 (40 mg, 6 equiv.) at 37 °C (24 h). The product was isolated by purification using gradient reversed phase chromatography (C-18, 12 g) eluting with water-MeOH (5-95% gradient). **^{1}H NMR** (500 MHz, CD_3OD) : δ 10.17 (s, 1H), 7.58 (t, *J* = 4.5 Hz, 1H), 7.52 (d, *J* = 8.0 Hz, 2H), 7.32 (d, *J* = 7.9 Hz, 2H), 7.16 – 7.08 (m, 3H), 4.48 – 4.43 (m, 1H), 3.37 (dd, *J* = 14.5, 5.4 Hz, 1H), 3.17 (dd, *J* = 14.6, 7.6 Hz, 1H), 2.43 (s, 3H), 1.41 & 1.21 (2 × s, 7H+2H = 9H) ppm; **^{13}C NMR** (100 MHz, $CDCl_3$) : δ 176.6, 165.5, 155.6, 137.2, 136.0, 134.0, 129.9, 128.1, 125.7, 123.2, 122.0, 120.2, 110.4, 80.3, 54.2, 28.3, 27.6, 21.2 ppm; **MS (ESI)** *m/z* 417.17 $[M+Na]^+$, 811.35 $[2M+Na]^+$; **HRMS (FTMS +p ESI)** *m/z* $C_{23}H_{26}N_2O_4Na$ $[M+Na]^+$ calculated 417.1785, found 417.1782.

6a: 1-((4'-methyl-[1,1'-biphenyl]-2-yl)diphenylmethyl)-1H-imidazole (from clotrimazole).

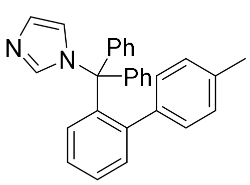

The general procedure afforded 34.8 mg (87% isolated yield) of the desired product as a white crystalline solid. ^{1}**H NMR** (400 MHz, $CDCl_3$) : δ 7.85 (d, *J* = 7.5 Hz, 2H), 7.77 (s, 1H), 7.46 (dd, *J* = 7.9, 1.5 Hz, 1H), 7.43 – 7.34 (m, 7H), 7.29 (d, *J* = 7.0 Hz, 1H), 7.25 – 7.15 (m, 7H), 6.97 (dd, *J* = 8.0, 1.5 Hz, 1H), 6.78 (t, *J* = 1.5 Hz, 1H), 2.40 (s, 3H) ppm. ^{13}**C NMR** (126 MHz, $CDCl_3$) : δ 140.1, 139.8, 138.7, 138.2, 135.5, 133.6, 132.3, 130.4, 130.1, 130.0, 128.4, 128.1, 128.1, 127.1, 121.8, 75.7, 21.6 ppm. **HRMS (FTMS +p NSI)** *m/z* $C_{29}H_{24}N_2H$ $[M+H]^+$ calculated for 401.2018, found 401.2019.

6b: 10-(3-(4-methylpiperazin-1-yl)propyl)-2-(p-tolyl)-10H-phenothiazine (from prochlorperazine).

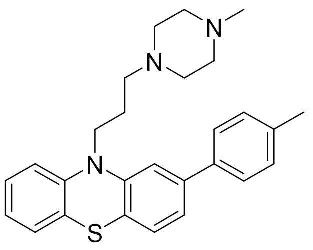

The general procedure afforded 38.6 mg (90% isolated yield) of the desired product as an orange viscous liquid. ^{1}**H NMR** (400 MHz, $CDCl_3$) : δ 7.43 (d, *J* = 8.1 Hz, 2H), 7.24 (d, *J* = 7.9 Hz, 2H), 7.19 – 7.08 (m, 4H), 7.05 (d, *J* = 1.6 Hz, 1H), 6.95 – 6.87 (m, 2H), 3.99 (t, *J* = 6.8 Hz, 2H), 2.72 – 2.45 (m, 6H), 2.40 (s, 6H), 2.28 (s, 4H), 1.99 (p, *J* = 7.0 Hz, 2H) ppm; ^{13}**C NMR** (126 MHz, $CDCl_3$) : δ 145.5, 145.1, 140.6, 138.0, 137.1, 129.4, 127.5, 127.4, 127.2, 126.8, 125.1, 123.9, 122.4, 121.2, 115.6, 114.3, 55.5, 54.8, 52.9, 45.7, 45.2, 24.3, 21.1 ppm; **HRMS (FTMS +p NSI)** *m/z* $C_{27}H_{32}N_3S$ $[M+H]^+$ calculated for 430.2311, found 430.2303.

6c: 2-(5-methoxy-2-methyl-1-(4'-methyl-[1,1'-biphenyl]-4-carbonyl)-1H-indol-3-yl)acetic acid (from indomethacine).

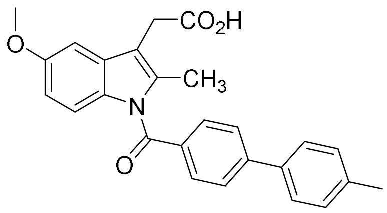

The general procedure afforded 30.4 mg (71% isolated yield) of the desired product as a yellowish solid. ^{1}**H NMR** (400 MHz, $CDCl_3$) : δ 7.78 (d, *J* = 8.4 Hz, 2H), 7.69 (d, *J* = 8.5 Hz, 2H), 7.56 (d, *J* = 8.2 Hz, 2H), 7.30 (d, *J* = 7.9 Hz, 2H), 7.06 – 6.86 (m, 2H), 6.67 (dd, *J* = 9.0, 2.6 Hz, 1H), 3.83 (s, 3H), 3.72 (s, 2H), 2.42 (s, 6H) ppm; ^{13}**C NMR** (101 MHz, $CDCl_3$) : δ 176.2, 169.2, 155.9, 145.6, 138.4, 136.7, 136.4, 133.7, 130.9, 130.5, 130.3, 129.7, 127.0, 115.1, 111.6, 111.3, 100.9, 55.7, 29.9, 21.2, 13.3 ppm; **HRMS (FTMS -p NSI)** *m/z* $C_{26}H_{22}NO_4$ $[M-H]^-$ calculated for 412.1554, found 412.1544. NMR data was in agreement with literature reported data. NMR data was in agreement with literature reported data.[12]

6e: 4-((4,4''-Dimethyl-[1,1':3',1''-terphenyl]-4'-yl)oxy)-4'-methyl-[1,1'-biphenyl]-3-ol (from irgasan).

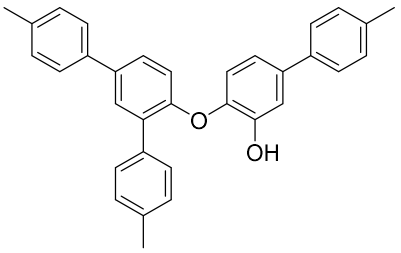

The general procedure afforded 39.9 mg (81% isolated yield) of the desired product as a white crystalline solid using *p*-tol-$B(OH)_2$ (4.5 equiv.) and K_2CO_3 (6 equiv.) after 66 h.^{1}**H NMR** (400 MHz, $CDCl_3$) : δ 7.67 (d, *J* = 2.3 Hz, 1H), 7.54 – 7.42 (m, 7H), 7.29 – 7.20 (m, 7H), 7.09 (d, *J* = 8.4 Hz, 1H), 7.04 (dd, *J* = 8.4, 2.2 Hz, 1H), 6.94 (d, *J* = 8.4 Hz, 1H), 5.56 (s, 1H), 2.40 (s, 3H), 2.38 (s, 6H) ppm; ^{13}**C NMR** (101 MHz, $CDCl_3$) : δ 152.3, 147.0, 143.7, 137.5, 137.4, 137.4, 137.3, 137.1, 136.9, 134.4, 133.6, 129.8, 129.5, 129.4, 129.1, 128.9, 126.9, 126.8, 126.7, 119.5, 118.9, 117.9, 114.4, 21.2, 21.1, 21.1 ppm; **HRMS (FTMS +p NSI)** *m/z* $C_{33}H_{32}O_2N$ $[M+NH_4]^+$ calculated for 474.2428, found 474.2420 ; $O_4H_{60}C_{66}N$ $[2M+NH_4]^+$ calculated for 930.4517, found 930.4516.

6f: 1-(4,4''-dimethyl-[1,1':2',1''-terphenyl]-4'-yl)-3-(4'-methyl-[1,1'-biphenyl]-4-yl)urea (from triclocarban).

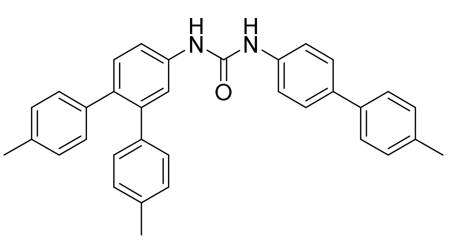

The general procedure afforded 39.5 mg (82% isolated yield) of the desired product as beige solid using *p*-tol-$B(OH)_2$ (4.5 equiv.) and K_2CO_3 (6 equiv.) after 66 h. ^{1}**H NMR** (400 MHz, DMSO-d_6) : δ 8.83 (d, *J* = 13.0 Hz, 2H), 7.62 – 7.43 (m, 8H), 7.31 – 7.21 (m, 3H), 7.02 (m, 8H), 2.33 (s, 3H), 2.27 (s, 3H), 2.25 (s, 3H) ppm; ^{13}**C NMR** (100 MHz, DMSO-d_6) : δ 152.5, 140.2, 138.9, 138.4, 138.1, 136.9, 136.0, 135.7, 135.2, 133.5, 133.5, 130.9, 129.5, 129.3, 129.2, 128.7, 128.6, 126.7, 125.9, 120.0, 118.6, 117.2, 20.7, 20.7 ppm;

HRMS (FTMS +p NSI) *m/z* $C_{34}H_{31}N_2O$ $[M+H]^+$ calculated for 483.2431, found 483.2421.

References:

1. M. J. Corr, S. V. Sharma, C. Pubill-Ulldemolins, R. T. Bown, P. Poirot, D. R. M. Smith, C. Cartmell, A. Abou Fayad and R. J. M. Goss, Sonogashira diversification of unprotected halotryptophans, halotryptophan containing tripeptides; and generation of a new to nature bromo-natural product and its diversification in water, *Chem. Sci.*, 2017, **8**, 2039-2046.
2. S. V. Sharma, X. Tong, C. Pubill-Ulldemolins, C. Cartmell, E. J. A. Bogosyan, E. J. Rackham, E. Marelli, R. B. Hamed and R. J. M. Goss, Living GenoChemetics by hyphenating synthetic biology and synthetic chemistry in vivo, *Nature Commun.*, 2017, **8**.
3. G. Miao, P. Ye, L. Yu and C. M. Baldino, Microwave-promoted Suzuki reactions of aryl chlorides in aqueous media, *J. Org. Chem.*, 2005, **70**, 2332-2334.
4. J. H. Kim, M. Eum, T. H. Kim and J. Y. Lee, A novel pyrrolocarbazole donor for stable and highly efficient thermally activated delayed fluorescent emitters, *Dyes Pigm.*, 2017, **136**, 529-534.
5. B. Karimi, D. Elhamifar, J. H. Clark and A. J. Hunt, Ordered Mesoporous Organosilica with Ionic-Liquid Framework: An Efficient and Reusable Support for the Palladium-Catalyzed Suzuki-Miyaura Coupling Reaction in Water, *Chem. - Eur. J.*, 2010, **16**, 8047-8053, S8047/8041-S8047/8018.
6. A. K. Cooper, D. K. Leonard, S. Bajo, P. M. Burton and D. J. Nelson, Aldehydes and ketones influence reactivity and selectivity in nickel-catalysed Suzuki-Miyaura reactions, *Chem. Sci.*, 2020, **11**, 1905-1911.
7. S. M. Raders, J. V. Kingston and J. G. Verkade, Advantageous Use of tBu2P-N=P(iBuNCH2CH2)3N in the Hiyama Coupling of Aryl Bromides and Chlorides, *J. Org. Chem.*, 2010, **75**, 1744-1747.
8. D. X. Yang, S. L. Colletti, K. Wu, M. Song, G. Y. Li and H. C. Shen, Palladium-Catalyzed Suzuki−Miyaura Coupling of Pyridyl-2-boronic Esters with Aryl Halides Using Highly Active and Air-Stable Phosphine Chloride and Oxide Ligands, *Org. Lett.*, 2009, **11**, 381-384.
9. K. Kumarasamy, T. Devendhiran, M.-C. Lin and C.-W. Chiu, Synthesis and physical property studies of cyclometalated Pt(II) and Pd(II) complexes with tridentate ligands containing pyrazole and pyridine groups, *Polyhedron*, 2020, **191**, 114799.
10. C. Liu, N. Han, X. Song and J. Qiu, A General and Highly Efficient Method for the Construction of Aryl-Substituted N-Heteroarenes, *Eur. J. Org. Chem.*, 2010, 5548-5551, S5548/5541-S5548/5523.
11. C. M. So, W. K. Chow, P. Y. Choy, C. P. Lau and F. Y. Kwong, Remarkably Effective Phosphanes Simply with a PPh2 Moiety: Application to Pd-Catalysed Cross-Coupling Reactions for Tetra-ortho-substituted Biaryl Syntheses, *Chem. - Eur. J.*, 2010, **16**, 7996-8001, S7996/7991-S7996/7204.
12. T. Zhou, S. Ma, F. Nahra, A. M. C. Obled, A. Poater, L. Cavallo, C. S. J. Cazin, S. P. Nolan and M. Szostak, [Pd(NHC)(μ-Cl)Cl]2: Versatile and Highly Reactive Complexes for Cross-Coupling Reactions that Avoid Formation of Inactive Pd(I) Off-Cycle Products, *iScience*, 2020, **23**, 101377.

NMR Spectra

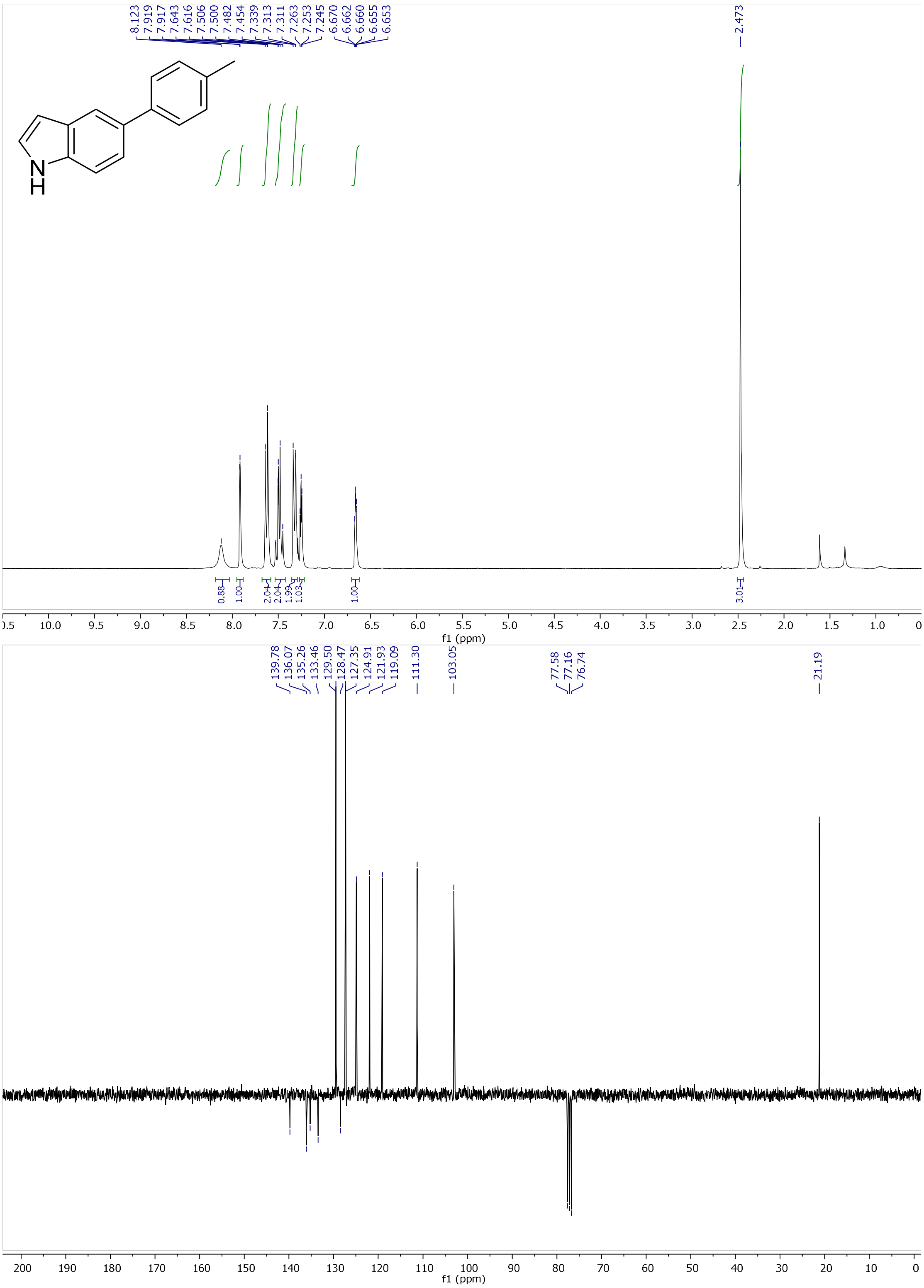

^{1}H and ^{13}C NMR of X1 5-(*p*-tolyl)-1H-indole (2a)

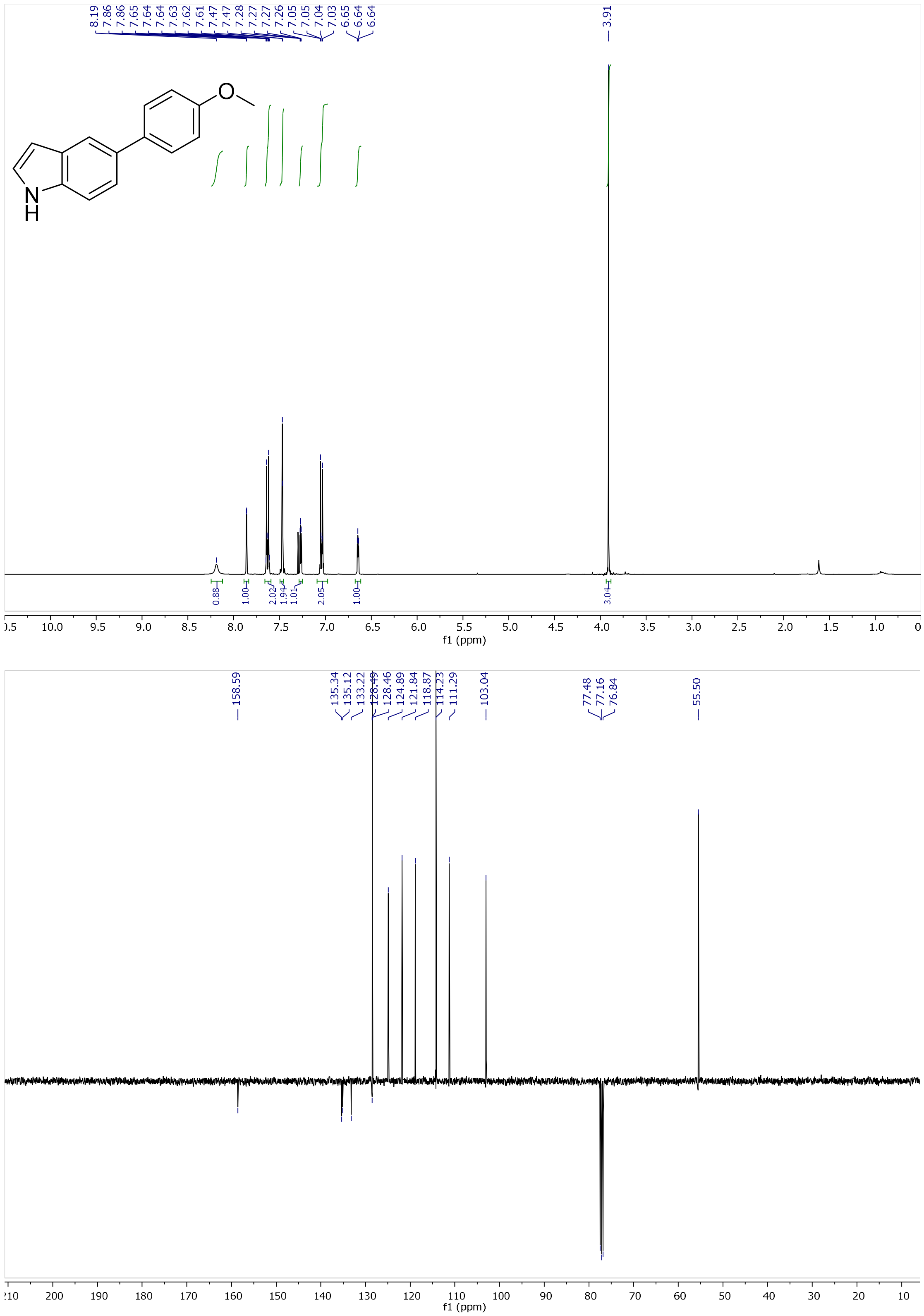

^{1}H and ^{13}C NMR of 5-(*p*-methoxyphenyl)-1H-indole (2b)

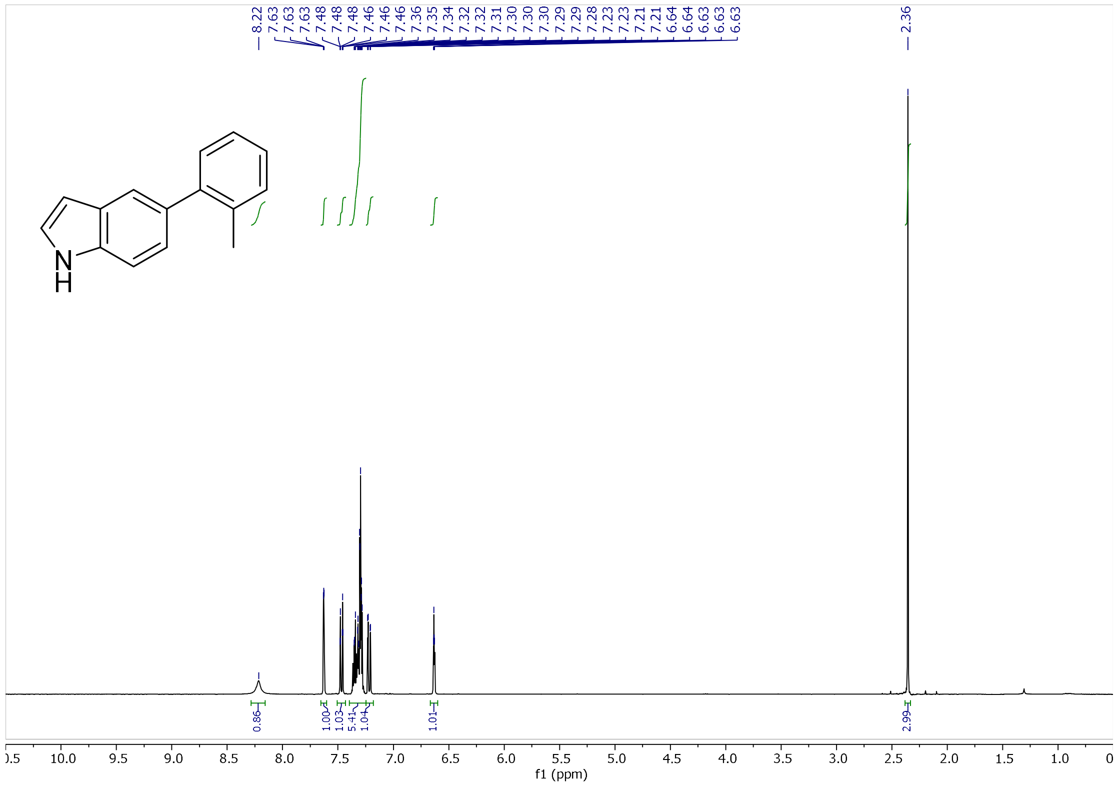

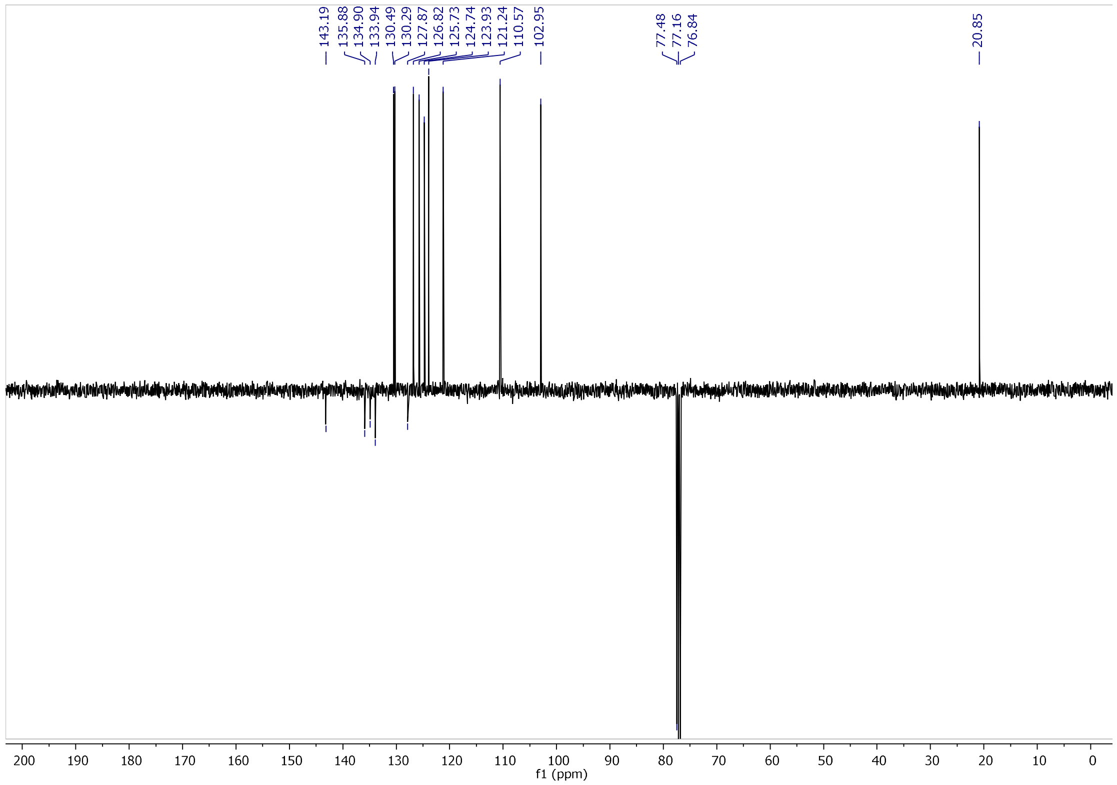

^{1}H and ^{13}C NMR of 5-(*o*-tolyl)-1H-indole (2c)

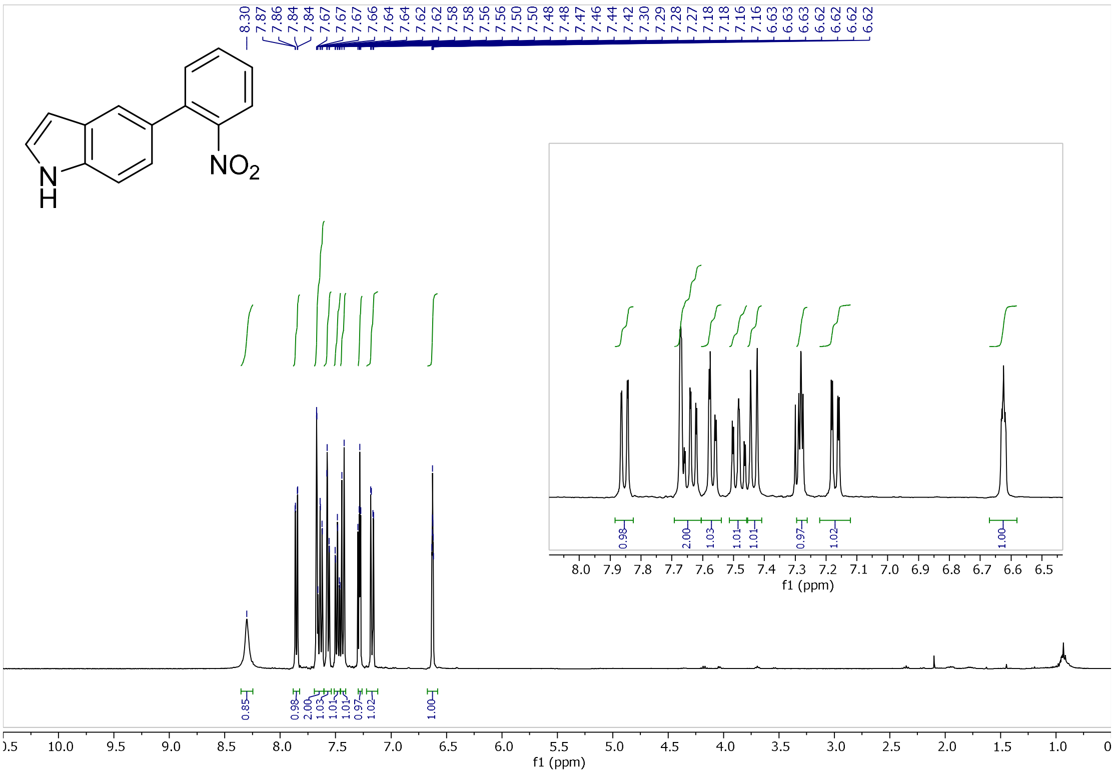

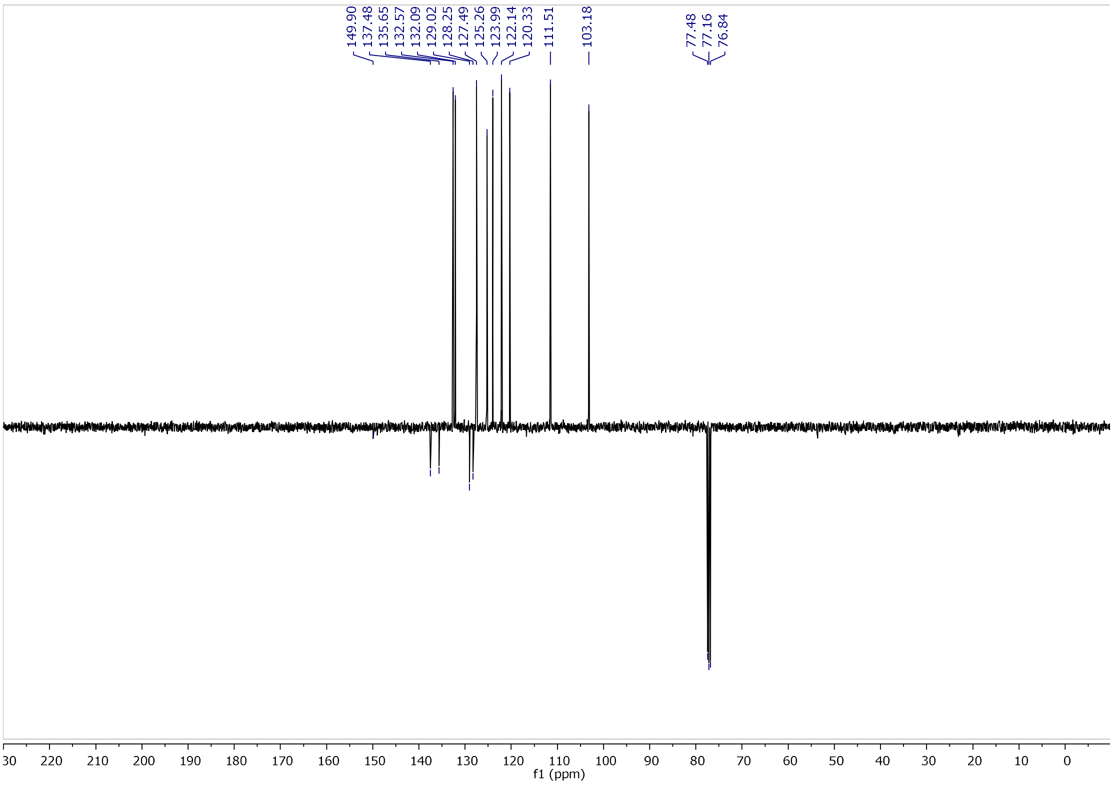

^{1}H and ^{13}C NMR 5-(*o*-nitrophenyl)-1H-indole (2d)

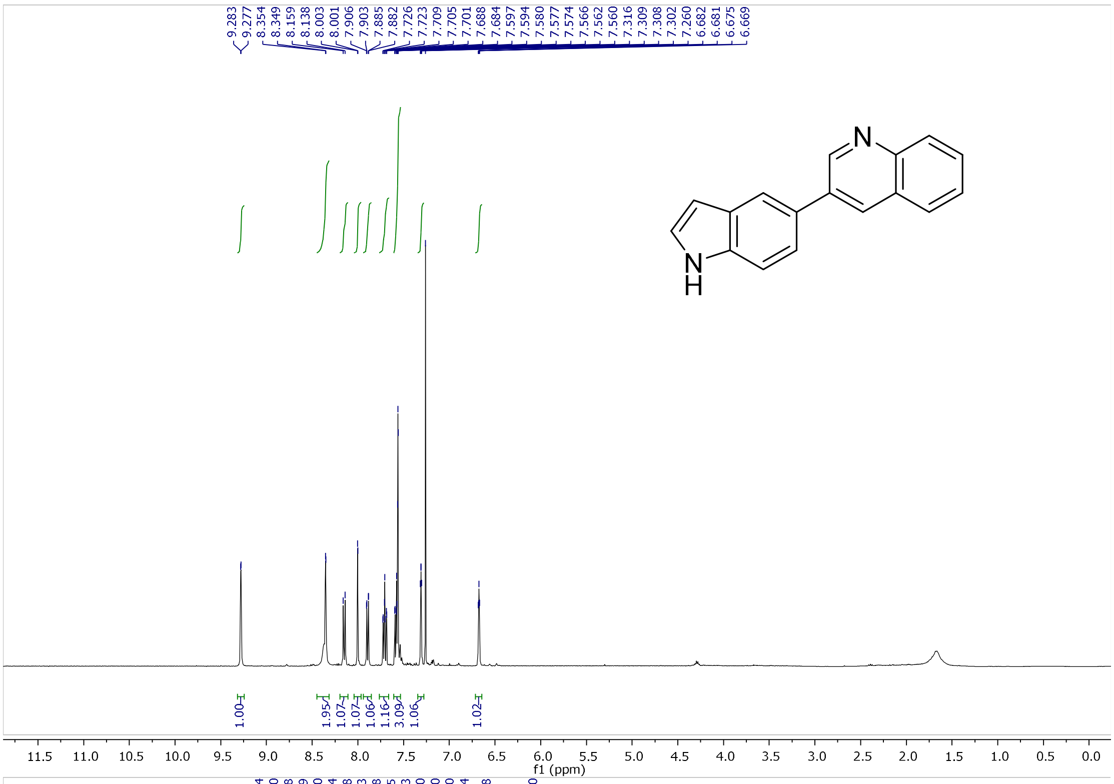

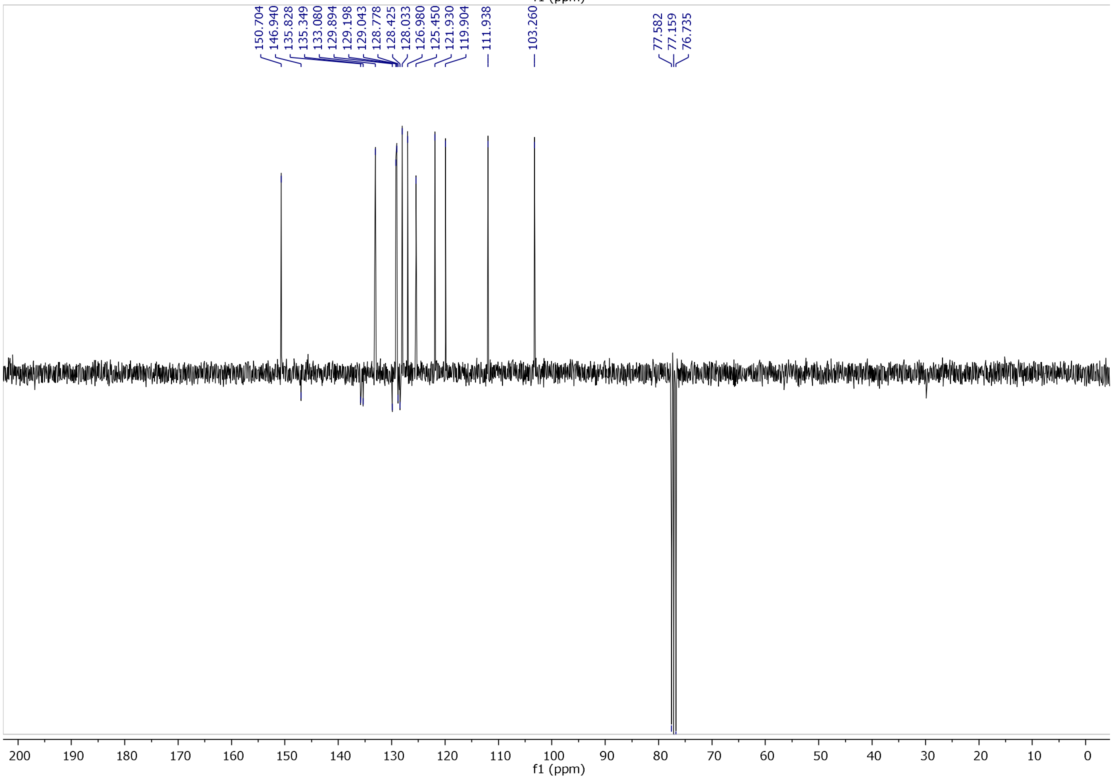

¹H and ¹³C NMR of 5-(quinolin-3-yl)-1H-indole (2e)

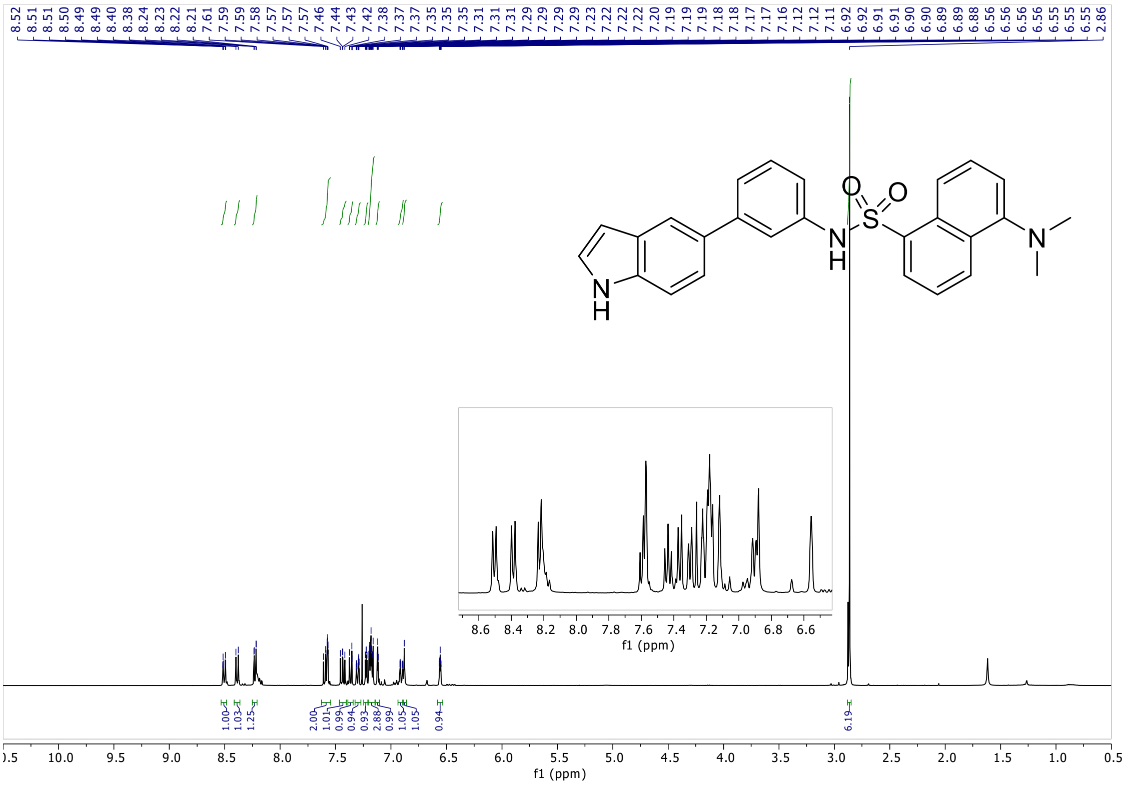

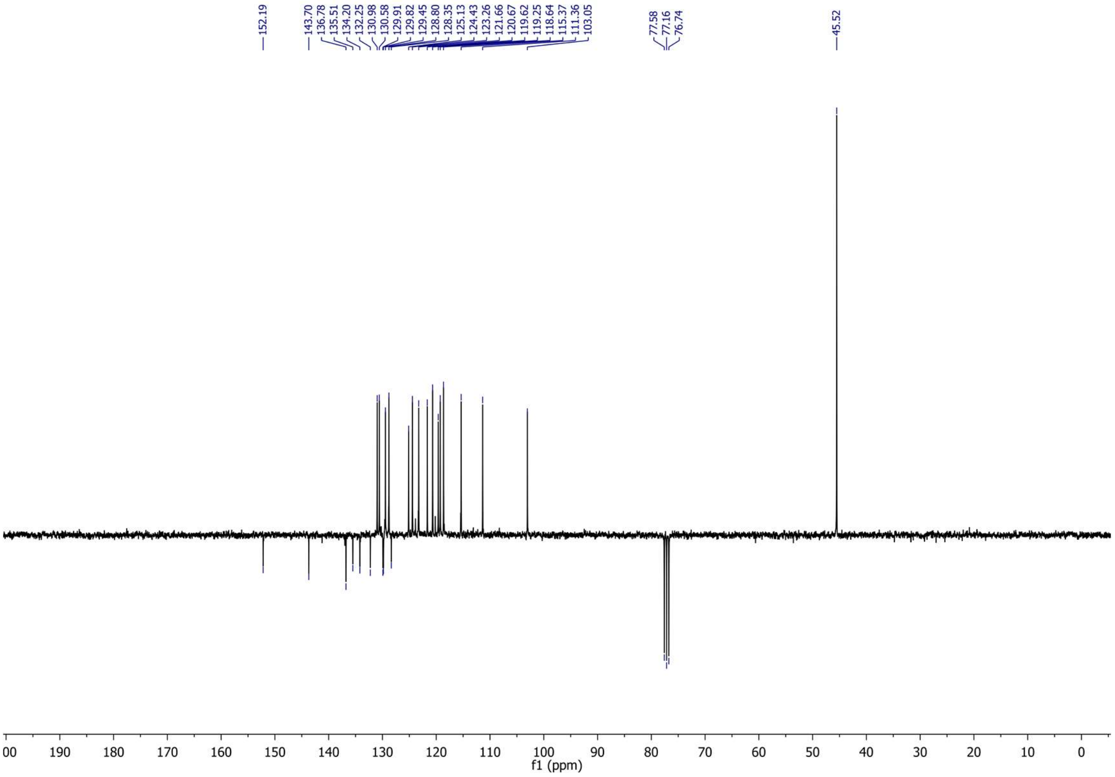

^{1}H and ^{13}C NMR of 5-[3-(5-dimethylaminonaphthalene-1-sulfonylamino)]-1H-indole (2f)

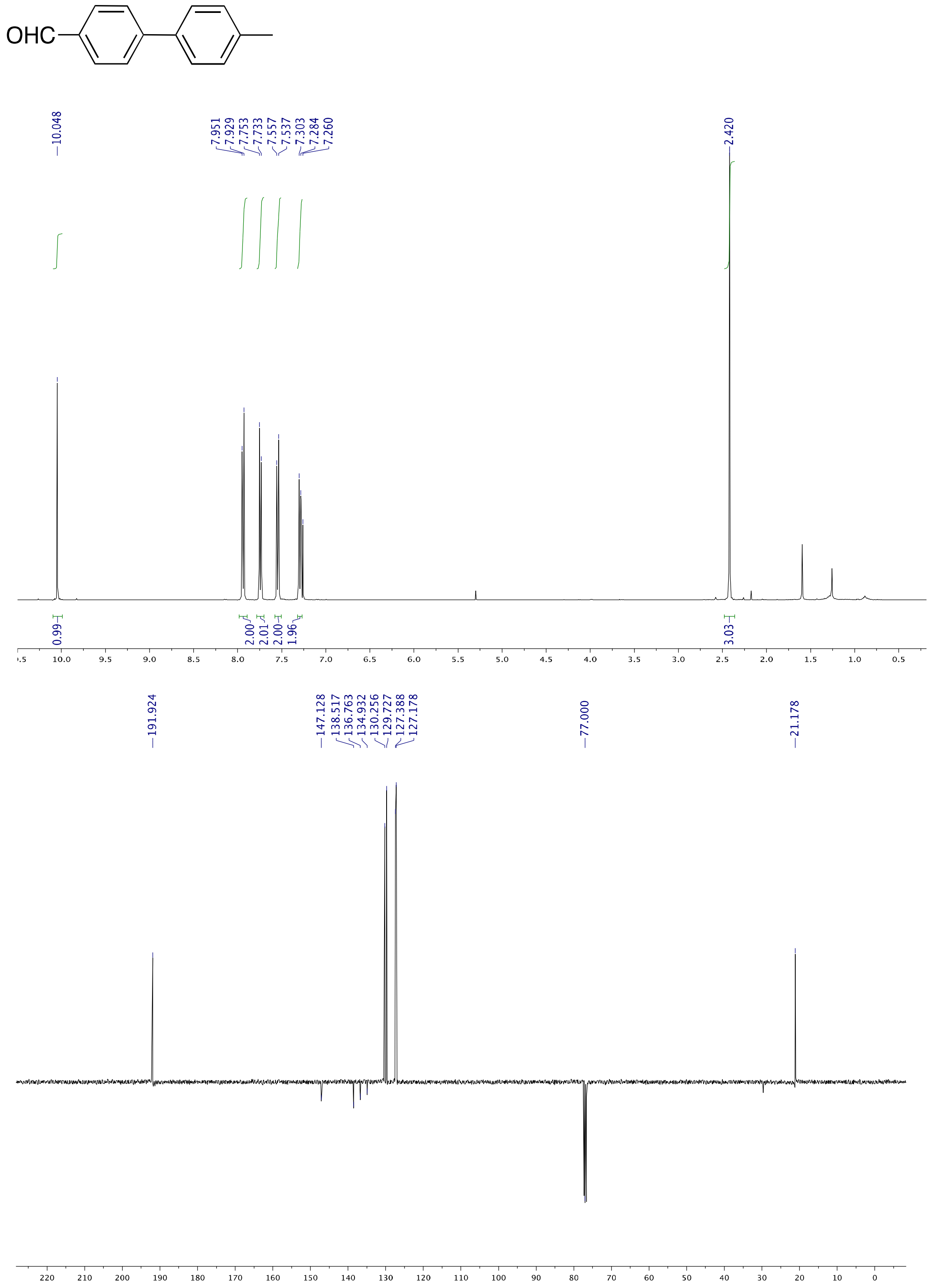

^{1}H and ^{13}C NMR of 4'-methyl-[1,1'-biphenyl]-4-carbaldehyde (4a)

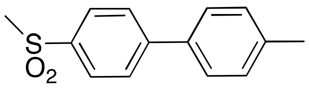

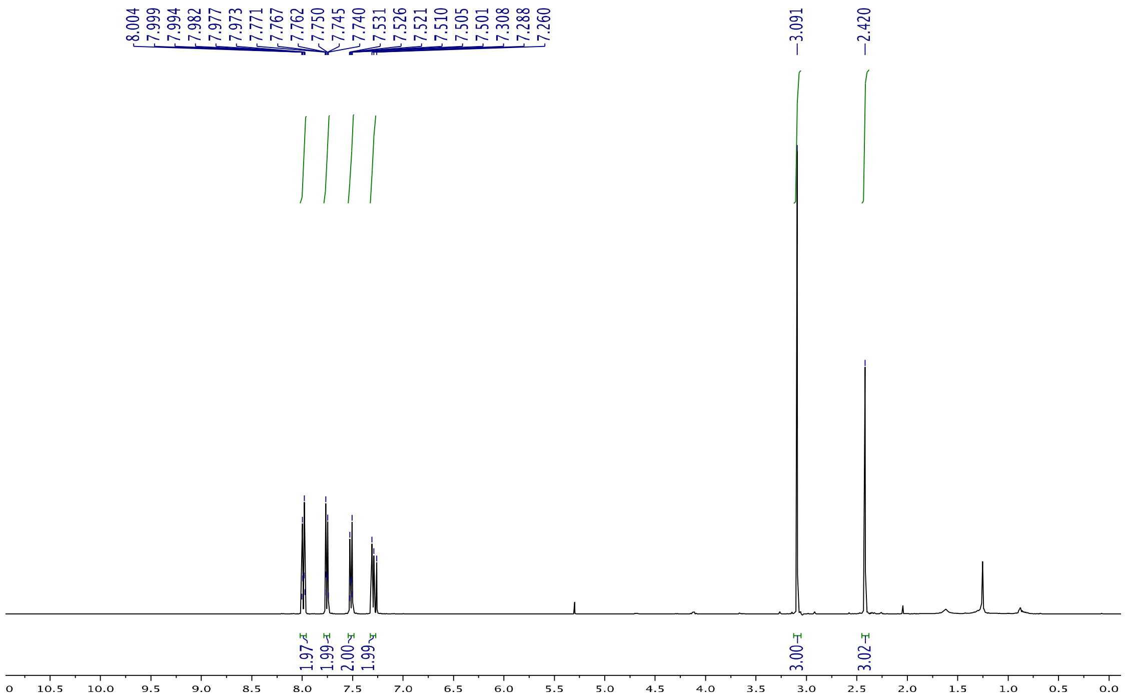

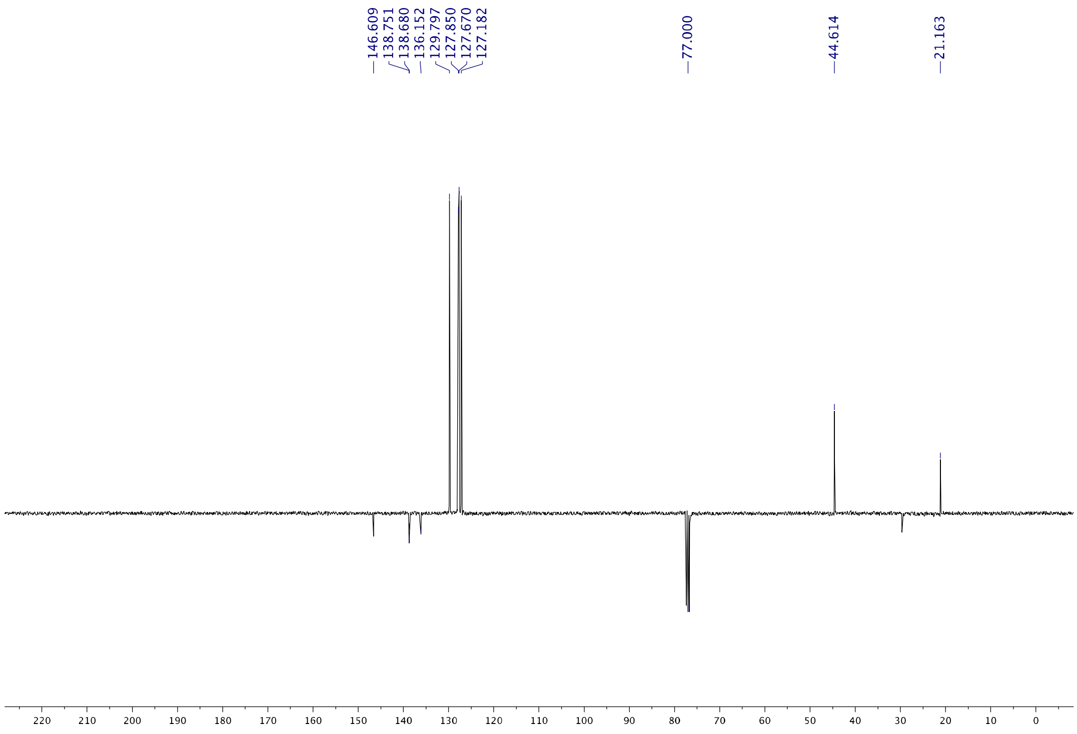

^{1}H and ^{13}C NMR of 4-methyl-4'-(methylsulfonyl)-1,1'-biphenyl (4b)

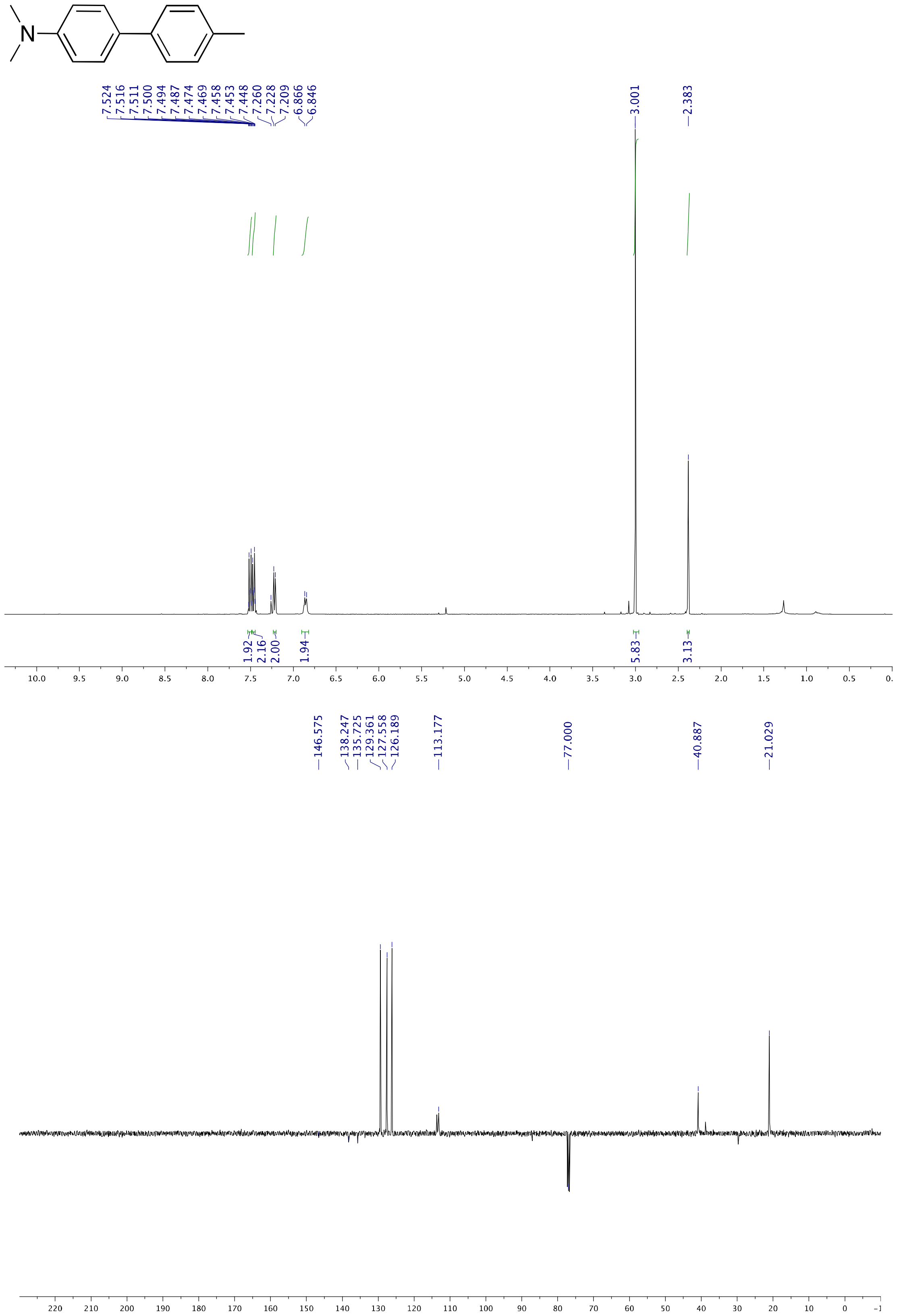

^{1}H and ^{13}C NMR of *N*,*N*,4'-trimethyl-[1,1'-biphenyl]-4-amine (4c)

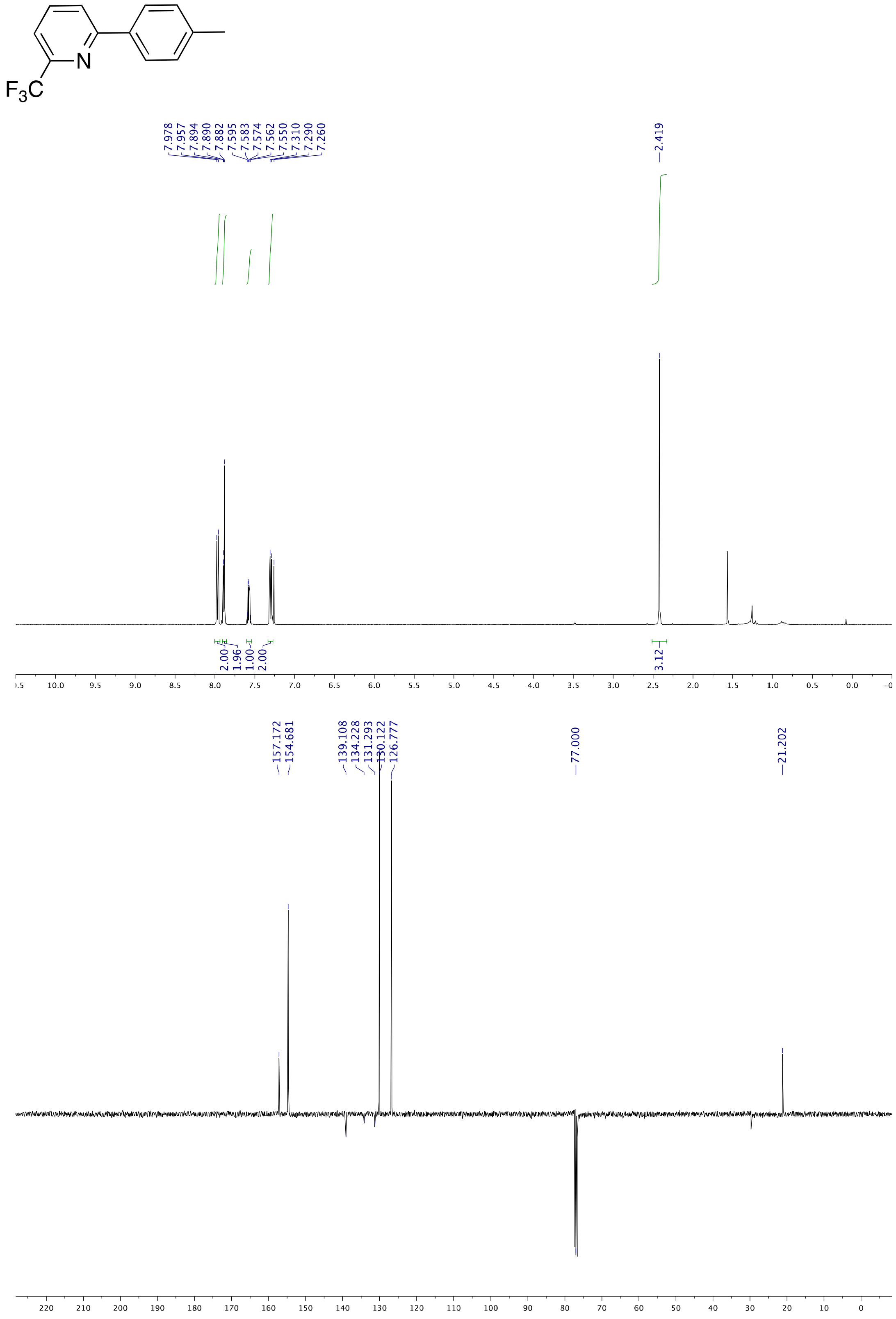

^{1}H and ^{13}C NMR of 2-(*p*-tolyl)-6-(trifluoromethyl)pyridine (4d)

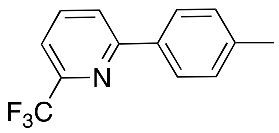

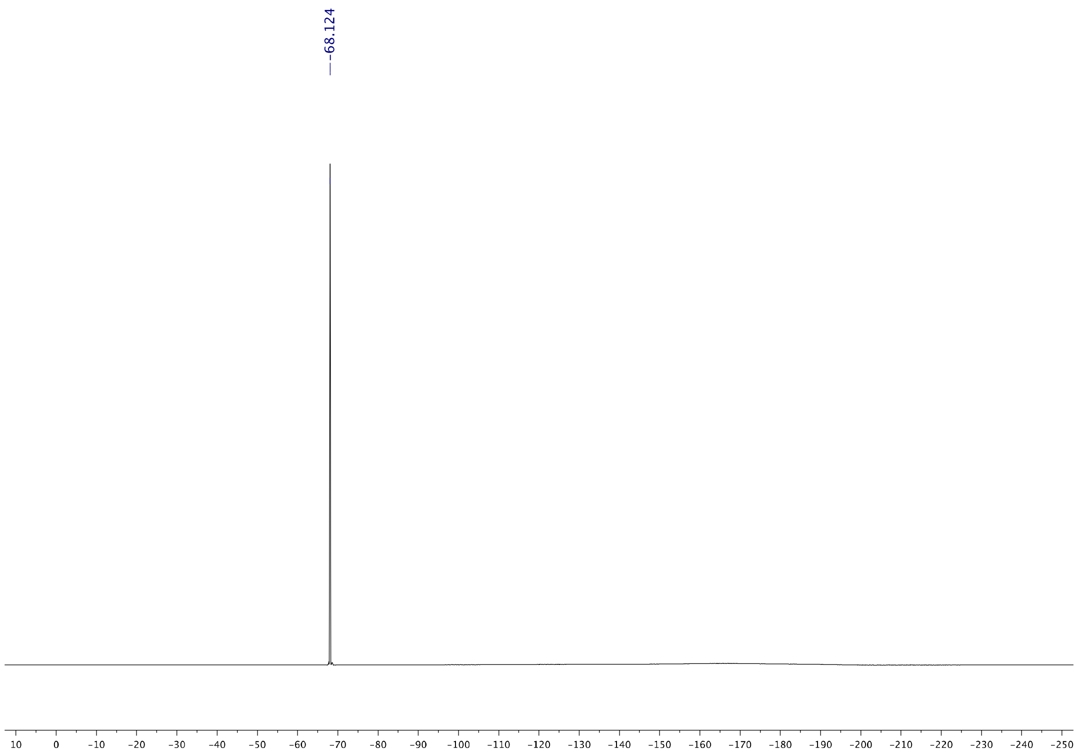

^{19}F NMR of 2-(*p*-tolyl)-6-(trifluoromethyl)pyridine (4d)

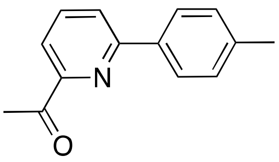

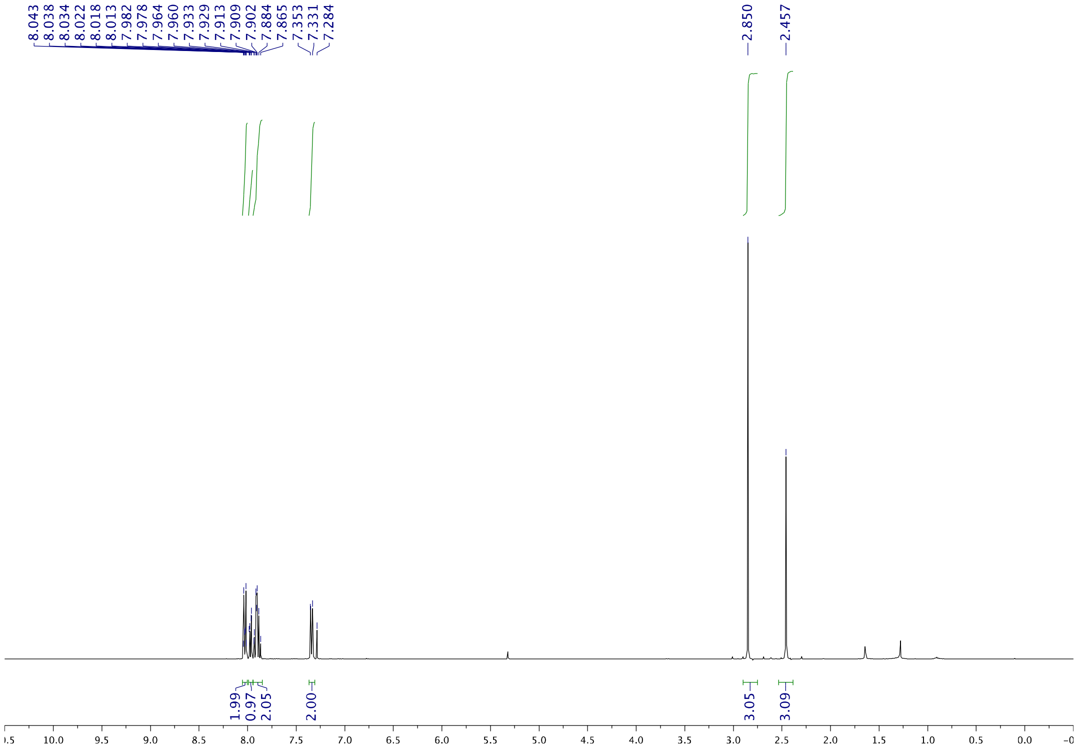

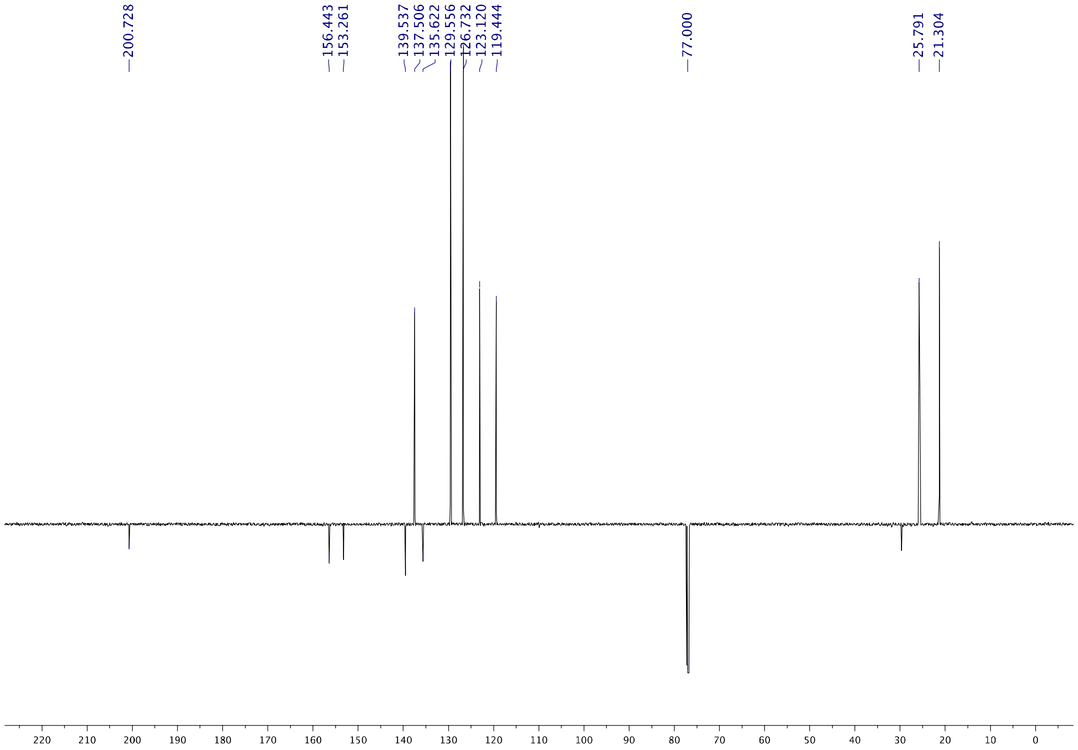

^{1}H and ^{13}C NMR of 1-(6-(*p*-tolyl)pyridin-2-yl)ethenone (4e)

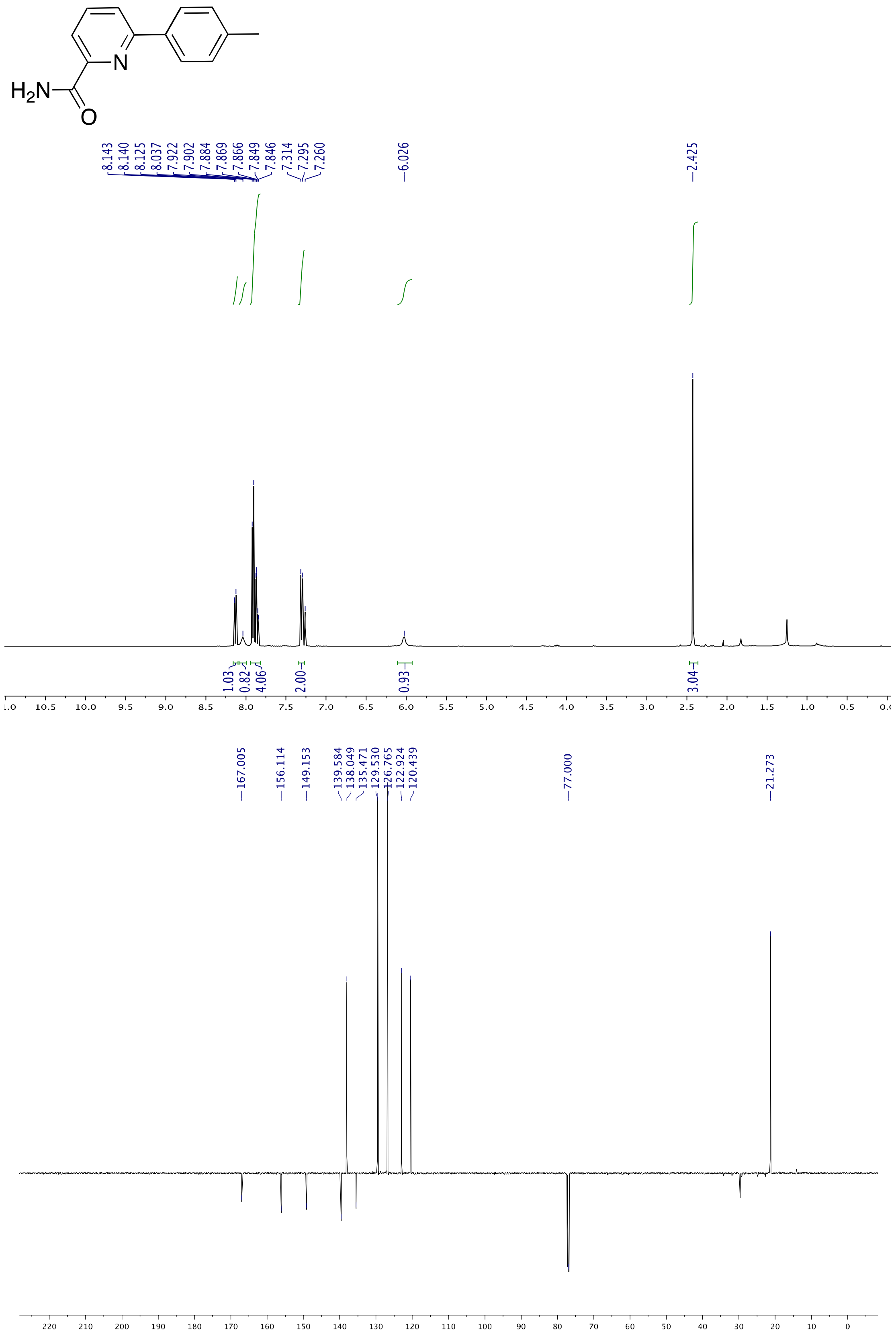

^{1}H and ^{13}C NMR of 6-(*p*-tolyl)picolinamide (4f)

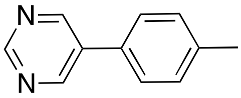

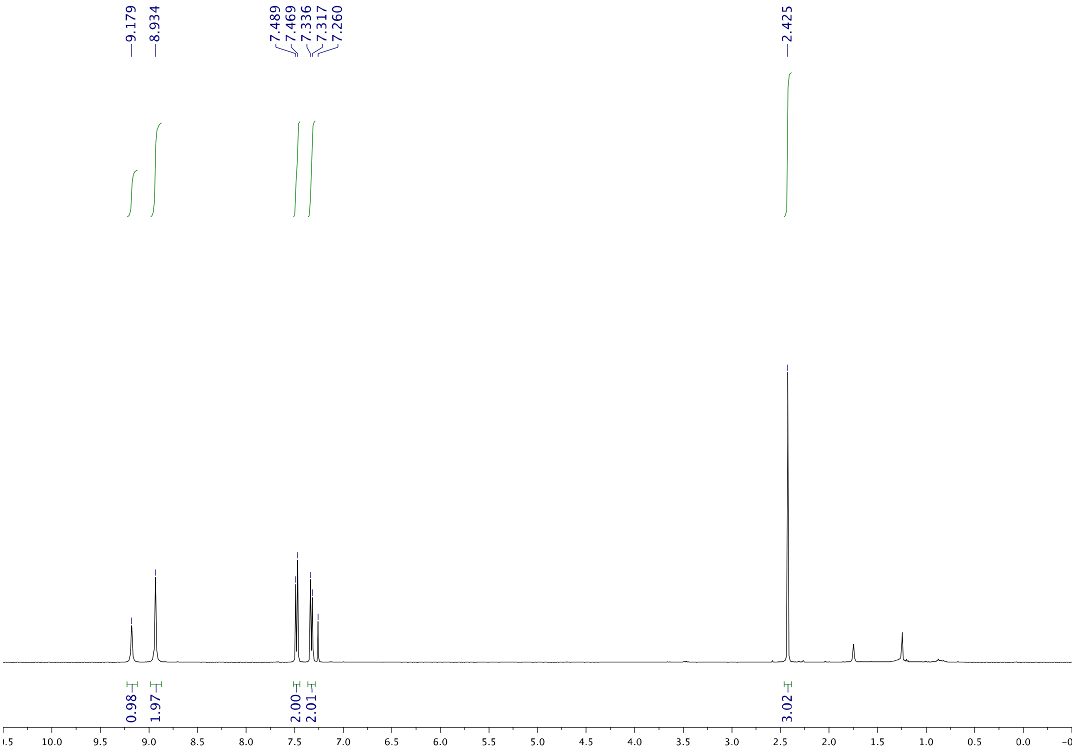

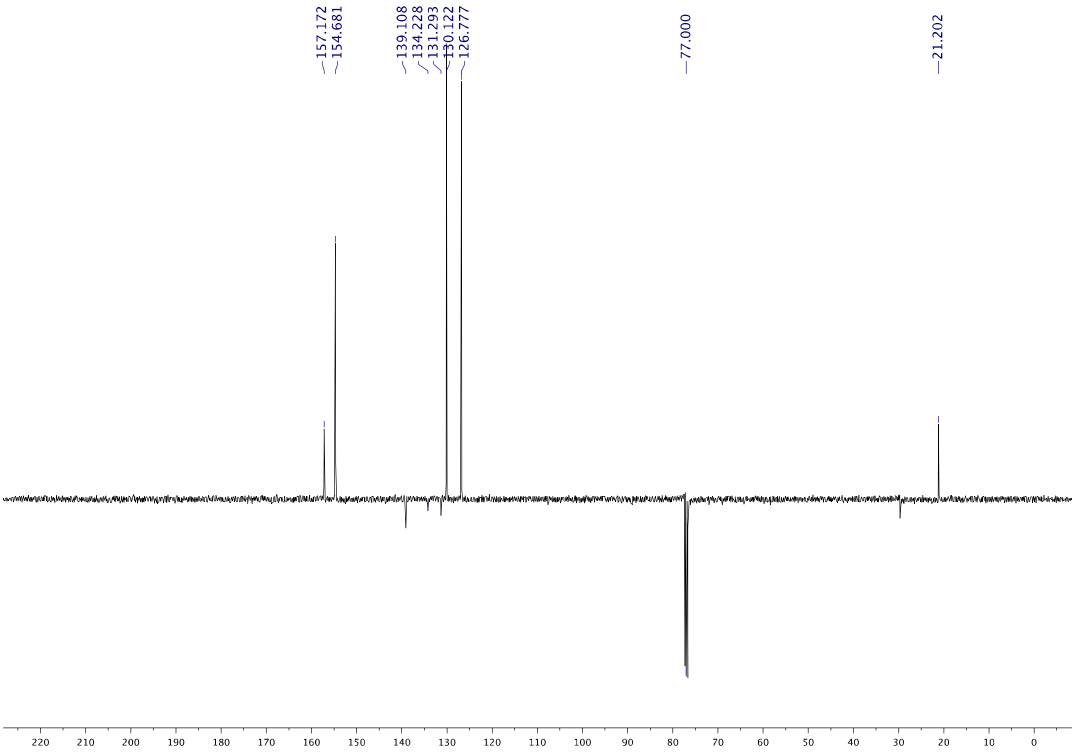

^{1}H and ^{13}C NMR of 5-(*p*-tolyl)pyrimidine (4g)

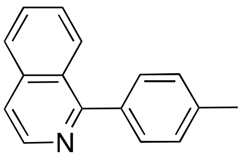

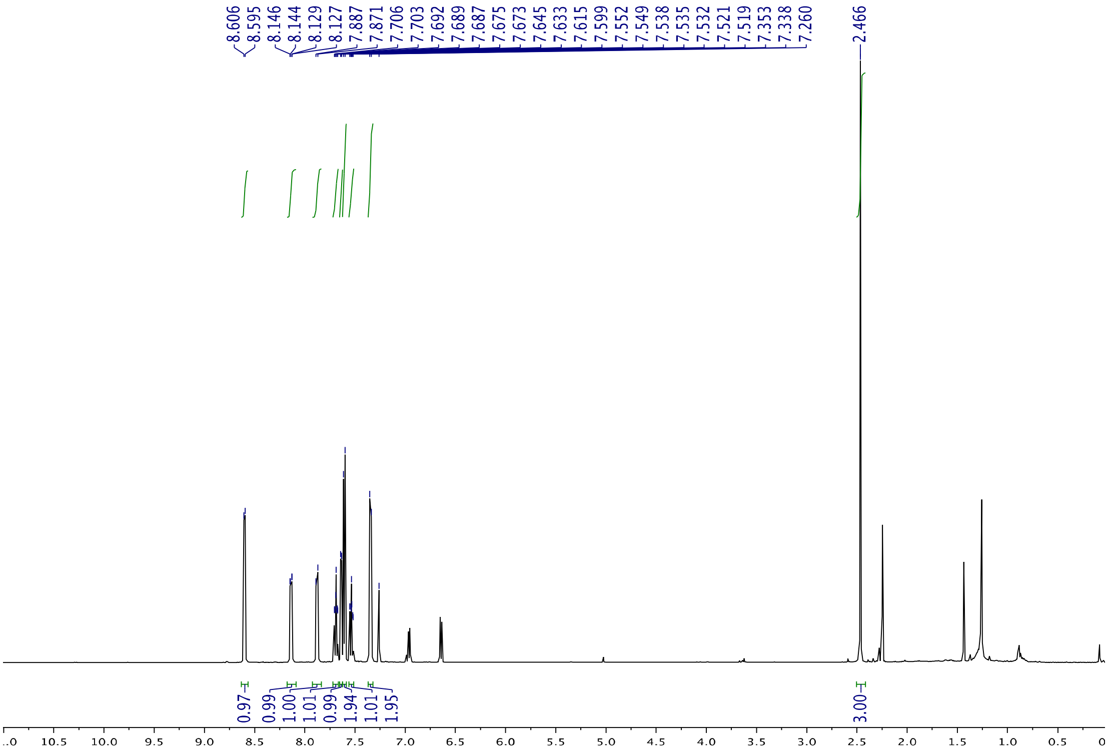

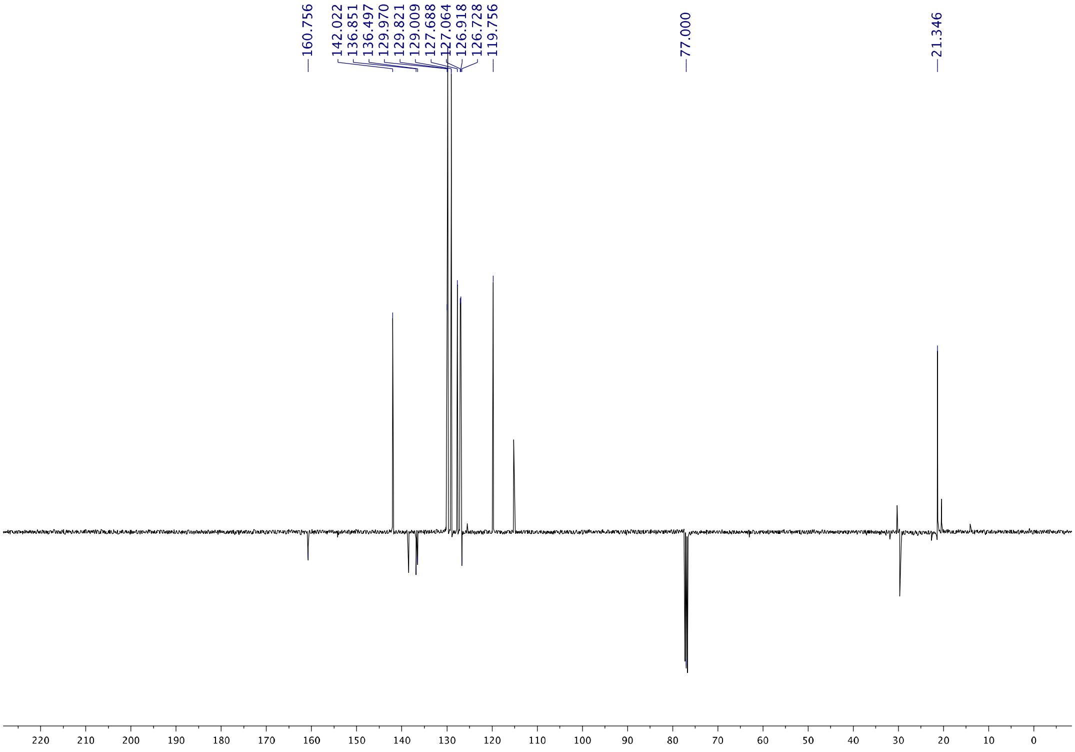

^{1}H and ^{13}C NMR of 1-(*p*-tolyl)isoquinoline (4h)

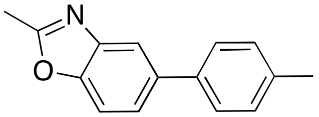

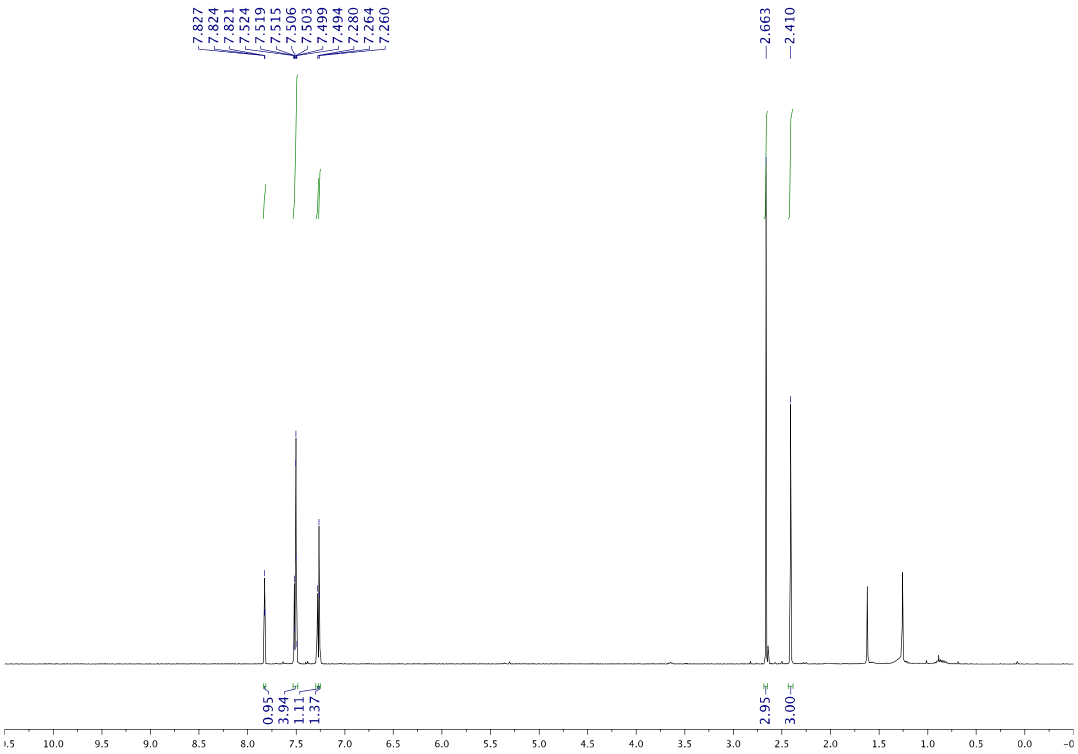

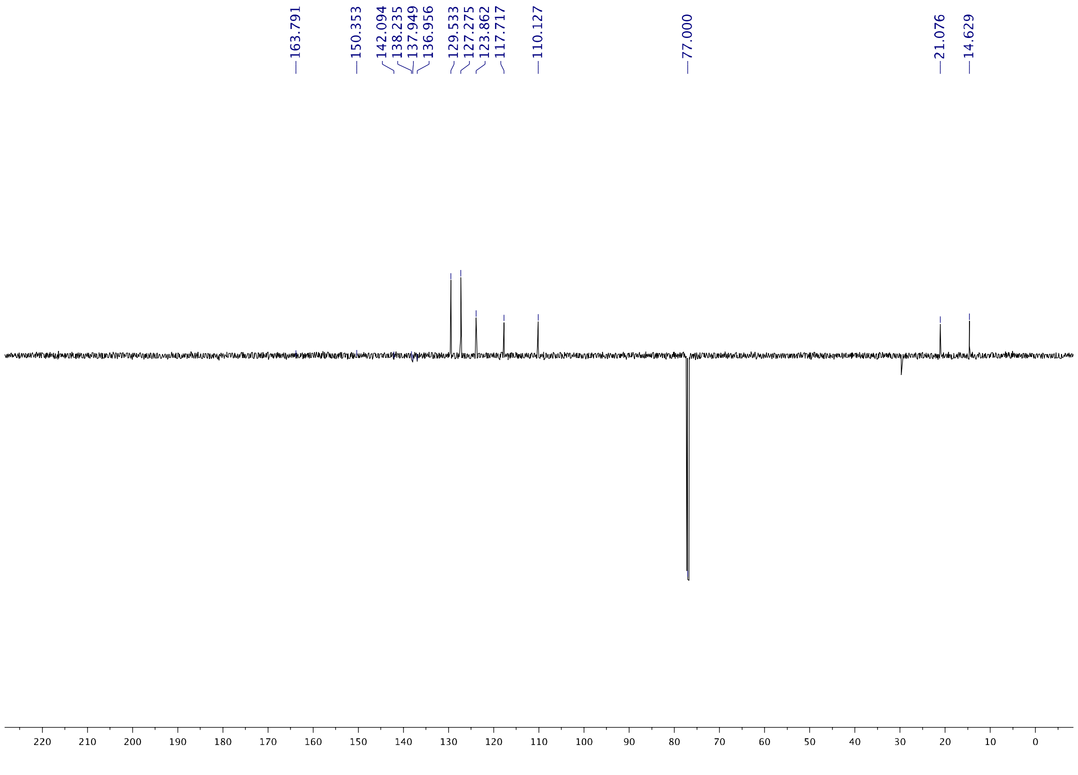

^{1}H and ^{13}C NMR of 2-methyl-5-(*p*-tolyl)benzo[*d*]oxazole (4i)

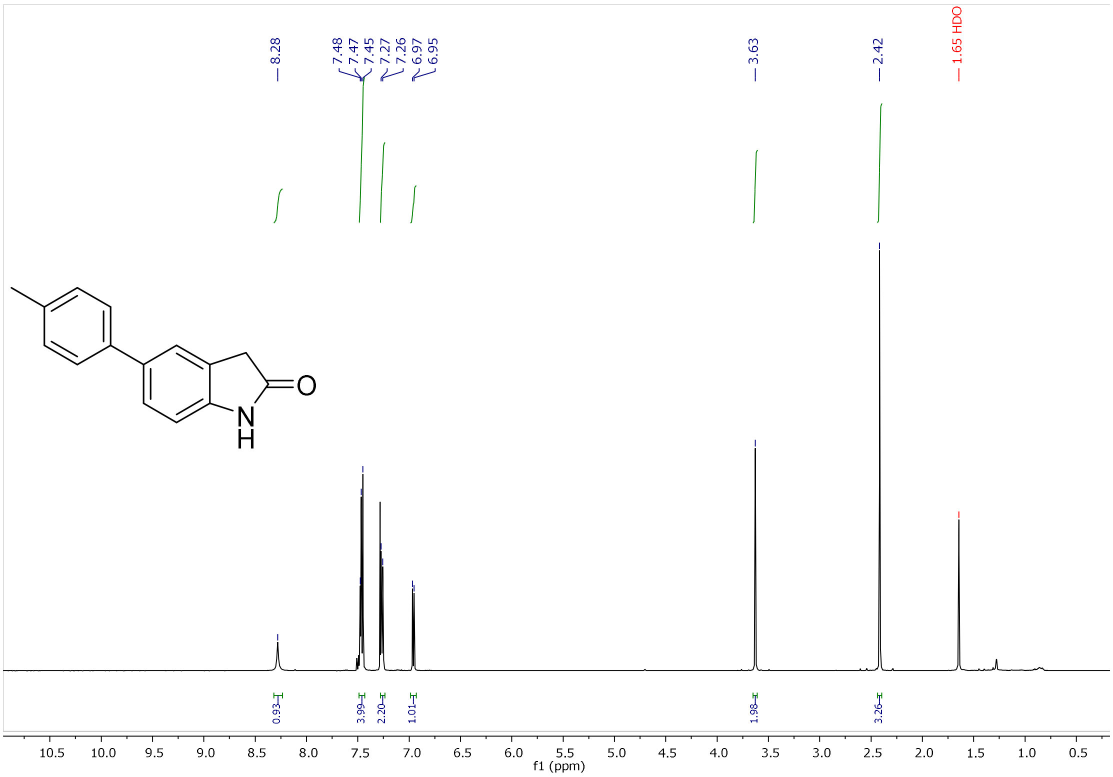

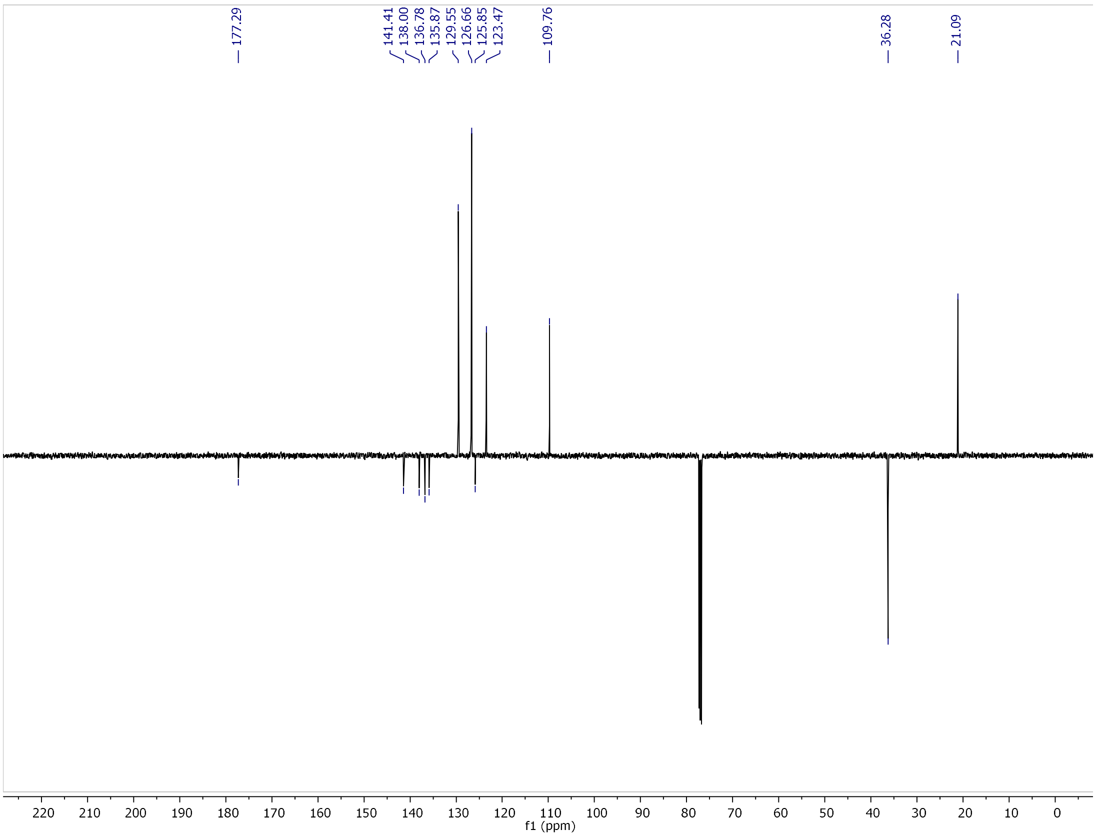

^{1}H and ^{13}C NMR of 5-(*p*-tolyl)-2-oxindole (4j)

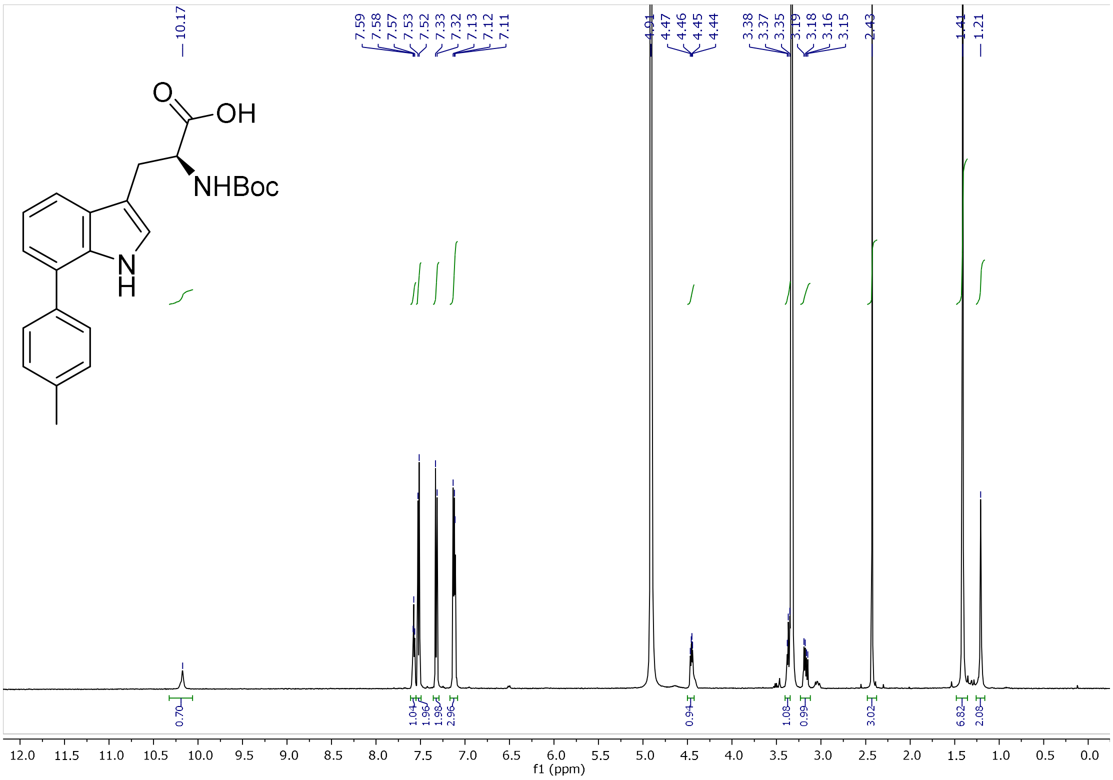

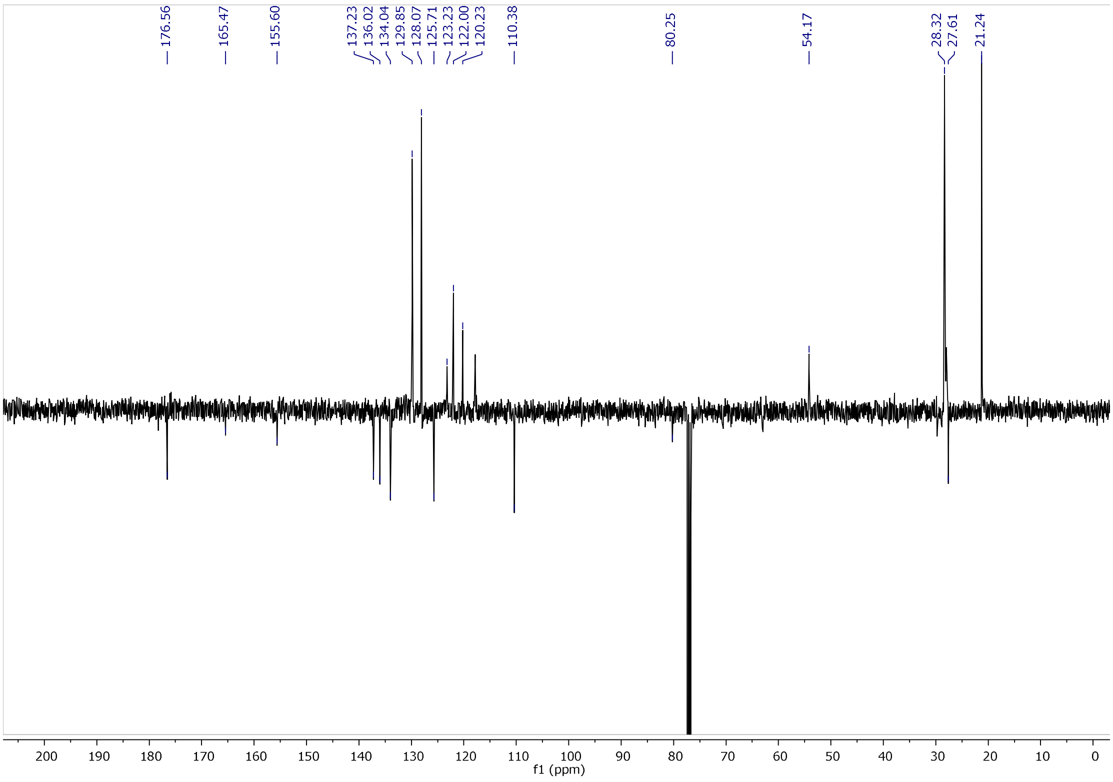

^{1}H and ^{13}C NMR of *N*-Boc-7-(*p*-tolyl)-*S*-tryptophan (4k)

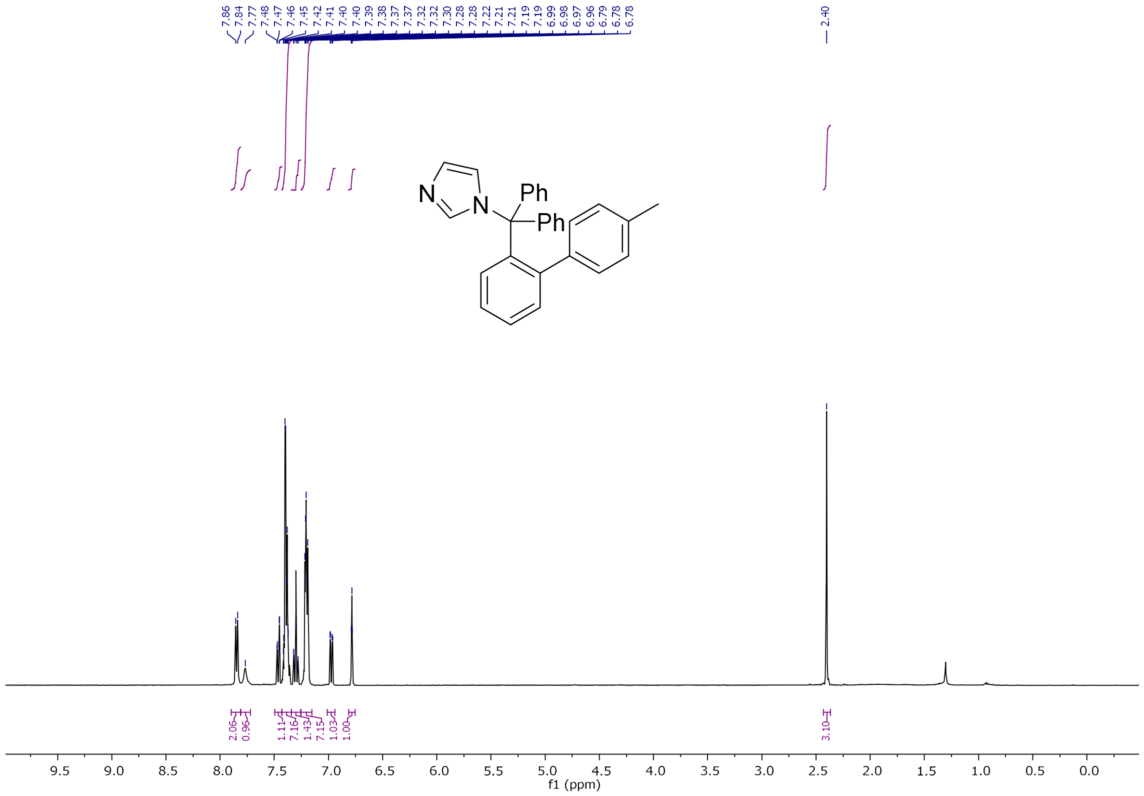

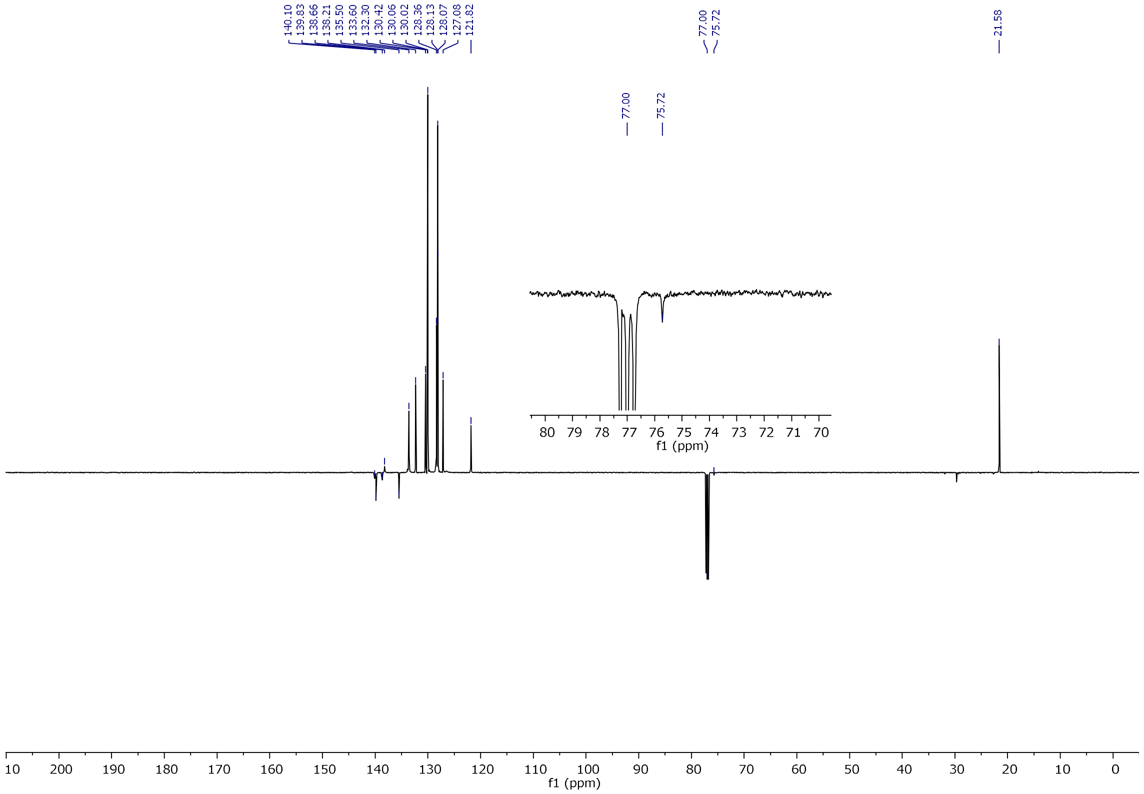

^{1}H and ^{13}C NMR of 1-((4'-methyl-[1,1'-biphenyl]-2-yl)diphenylmethyl)-1H-imidazole (6a)

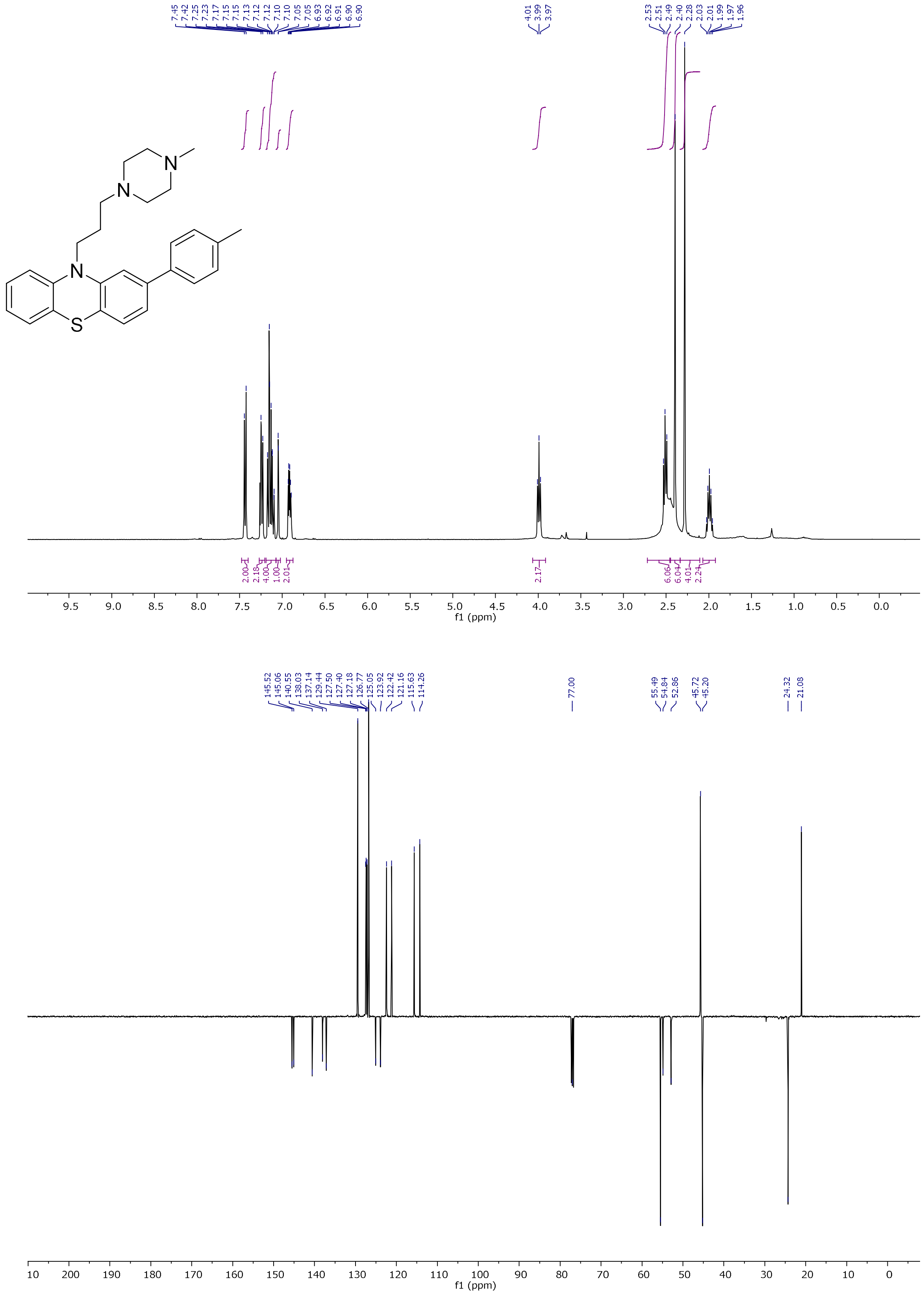

^{1}H and ^{13}C NMR of 10-(3-(4-methylpiperazin-1-yl)propyl)-2-(p-tolyl)-10H-phenothiazine (6b)

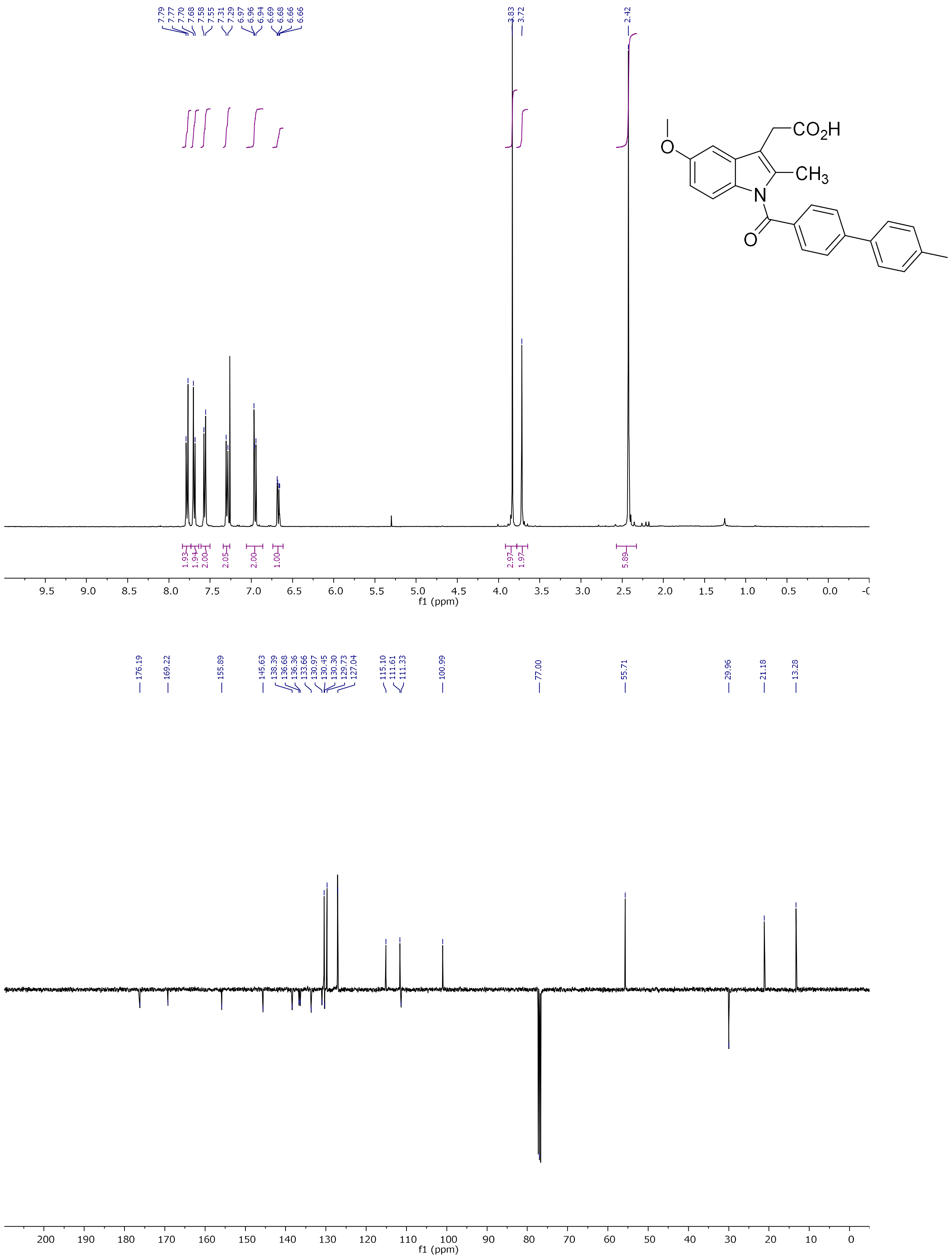

^{1}H and ^{13}C NMR of 2-(5-methoxy-2-methyl-1-(4'-methyl-[1,1'-biphenyl]-4-carbonyl)-1H-indol-3-yl)acetic acid (6c)

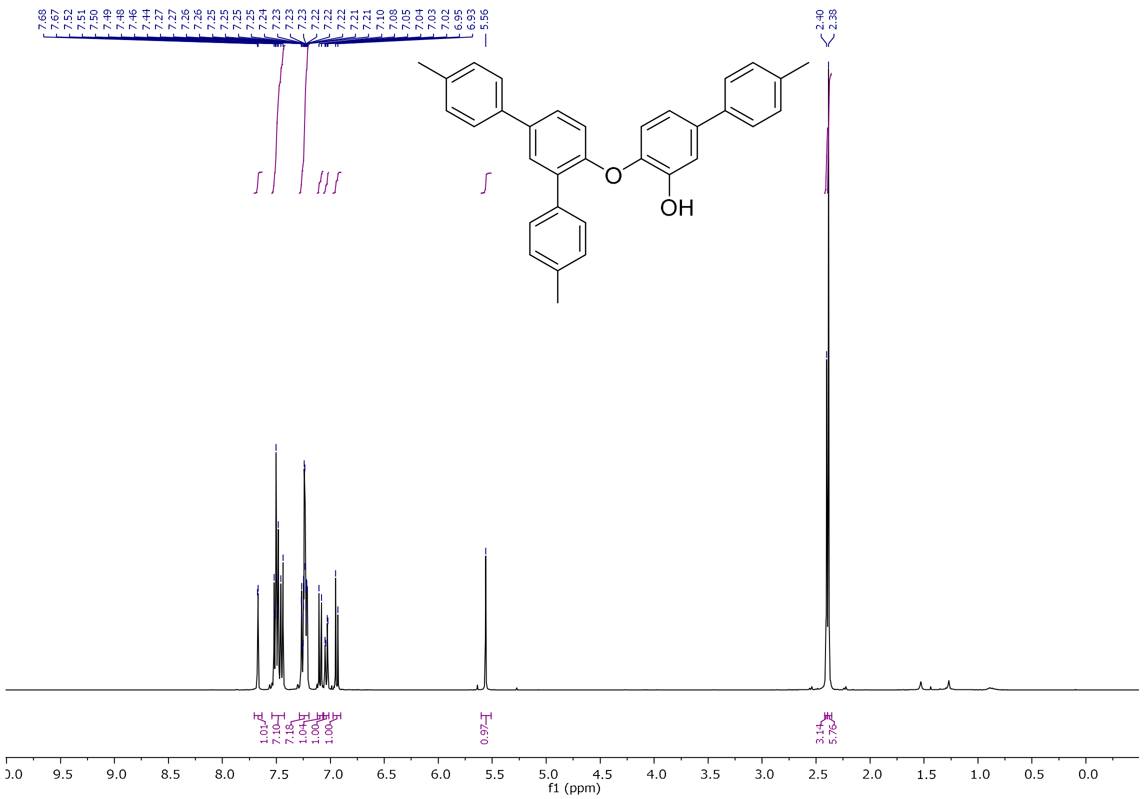

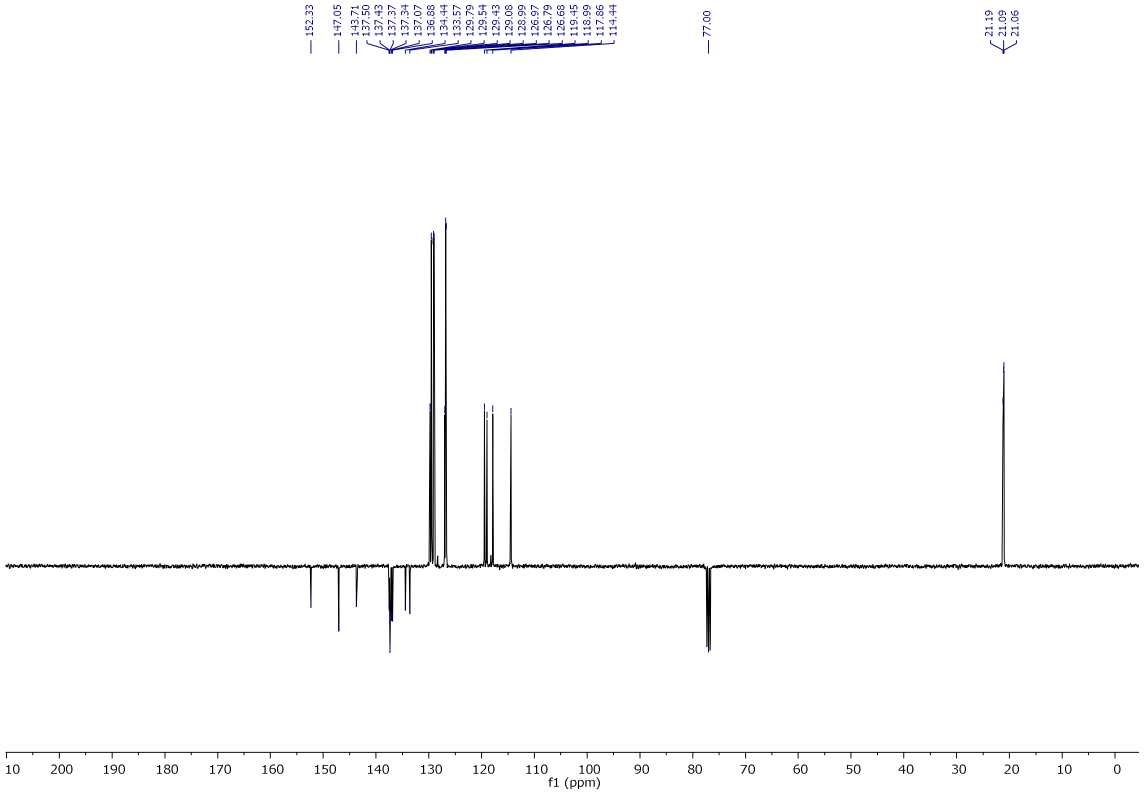

^{1}H and ^{13}C NMR of 4-((4,4''-dimethyl-[1,1':3',1''-terphenyl]-4'-yl)oxy)-4'-methyl-[1,1'-biphenyl]-3-ol (6e)

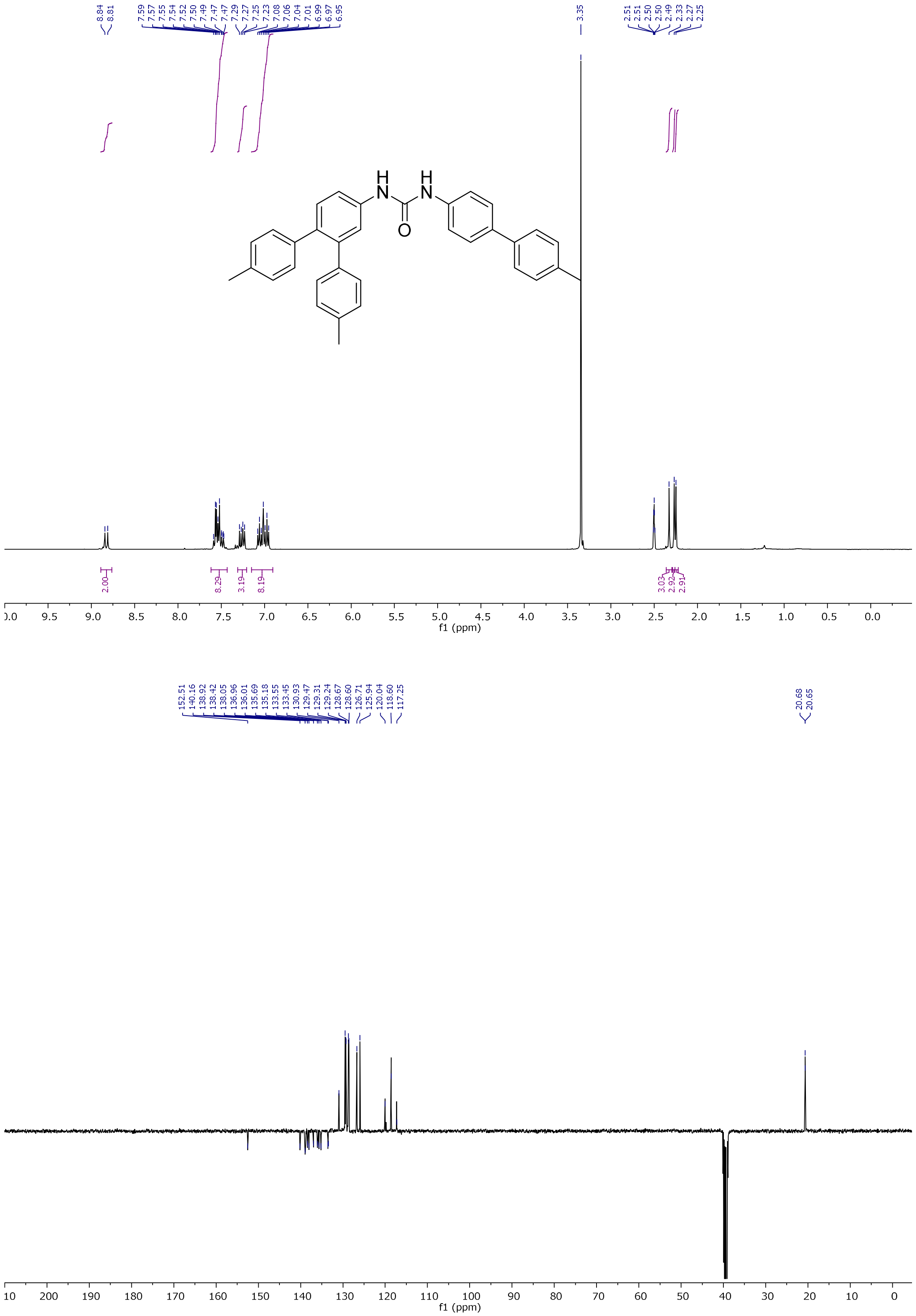

^{1}H and ^{13}C NMR of 1-(4,4''-dimethyl-[1,1':2',1''-terphenyl]-4'-yl)-3-(4'-methyl-[1,1'-biphenyl]-4-yl)urea (6f)
